# A Mutual Support Mechanism through Intercellular Movement of CAPRICE and GLABRA3 Can Pattern the *Arabidopsis* Root Epidermis

**DOI:** 10.1371/journal.pbio.0060235

**Published:** 2008-09-23

**Authors:** Natasha Saint Savage, Tom Walker, Yana Wieckowski, John Schiefelbein, Liam Dolan, Nicholas A. M Monk

**Affiliations:** 1 Department of Molecular Biology and Biotechnology, University of Sheffield, Sheffield, United Kingdom; 2 Department of Cell and Developmental Biology, John Innes Centre, Norwich, United Kingdom; 3 Department of Molecular, Cell, and Developmental Biology, University of Michigan, Ann Arbor, Michigan, United States of America; 4 Division of Applied Mathematics, School of Mathematical Sciences, University of Nottingham, Nottingham, United Kingdom; 5 Centre for Plant Integrative Biology, School of Biosciences, University of Nottingham, Loughborough, United Kingdom; Max Planck Institute for Developmental Biology, Germany

## Abstract

The patterning of the *Arabidopsis* root epidermis depends on a genetic regulatory network that operates both within and between cells. Genetic studies have identified a number of key components of this network, but a clear picture of the functional logic of the network is lacking. Here, we integrate existing genetic and biochemical data in a mathematical model that allows us to explore both the sufficiency of known network interactions and the extent to which additional assumptions about the model can account for wild-type and mutant data. Our model shows that an existing hypothesis concerning the autoregulation of WEREWOLF does not account fully for the expression patterns of components of the network. We confirm the lack of WEREWOLF autoregulation experimentally in transgenic plants. Rather, our modelling suggests that patterning depends on the movement of the CAPRICE and GLABRA3 transcriptional regulators between epidermal cells. Our combined modelling and experimental studies show that WEREWOLF autoregulation does not contribute to the initial patterning of epidermal cell fates in the *Arabidopsis* seedling root. In contrast to a patterning mechanism relying on local activation, we propose a mechanism based on lateral inhibition with feedback. The active intercellular movements of proteins that are central to our model underlie a mechanism for pattern formation in planar groups of cells that is centred on the mutual support of two cell fates rather than on local activation and lateral inhibition.

## Author Summary

The patterning of the *Arabidopsis* root epidermis depends on a genetic regulatory network that operates within and between cells. Genetic studies have identified a number of key components of this network, but the functional logic of the network has remained unclear. In this work, we integrate genetic and biochemical data in a mathematical model that we use to explore both the sufficiency of known network interactions and the extent to which additional assumptions about the model can account for wild-type and mutant data. Our model shows that an existing hypothesis concerning the autoregulation of the transcription factor WEREWOLF does not account fully for observed expression patterns, and we confirm the absence of autoregulation experimentally in transgenic plants. We propose an alternative mechanism centred on the movement of transcriptional regulators between epidermal cells, and present experimental support for this mechanism. These movements underlie a novel mechanism for pattern formation in planar groups of cells, centred on mutual support of two cell fates rather than local activation and lateral inhibition.

## Abstract 3

Patterning of the root epidermis is shown to depend on mutual support of alternative cell fates rather than on local activation and lateral inhibition. Movement of proteins between cells is central to this mechanism.

## Introduction

**Figure 1:**
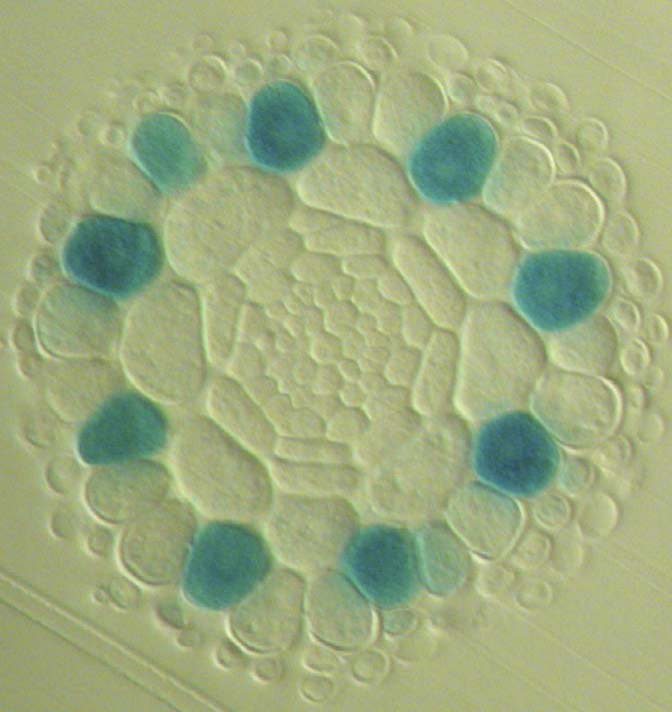
Cross-Section of an *Arabidopsis* Root The epidermis is the outer ring of large cells. Trichoblasts (marked by blue GUS staining) are located over the clefts between underlying cortical cells (the H position). Atrichoblasts (no staining) touch only one cortical cell (the N position). Note that trichoblasts are sometimes separated by more than one atrichoblast. This is a result of occasional anticlinal cell divisions in the epidermis, which increase the number of epidermal cells in a ring (newly formed epidermal rings in the apical meristem contain 16 cells).

The cells of the *Arabidopsis* root epidermis emerge from the initial cells in the root meristem with the potential to adopt either of two cell fates—trichoblasts (cells that can go on to differentiate as root hair cells) or atrichoblasts (that differentiate into non–hair-bearing epidermal cells). In the wild-type seedling, the two cell types are arranged in a stereotyped spatial pattern, with files of trichoblasts overlying two cortical cells (the H position) separated by files of atrichoblasts in contact with only one underlying cortical cell (the N position) (Figure 1) [1,2]. This fixed pattern does not result from lineage restriction, but depends on a combination of positional information from the cortex and the operation of a genetic regulatory network within the epidermis [3–5]. At the core of this network lie protein complexes centred on the basic helix-loop-helix proteins GLABRA3 (GL3) and ENHANCER OF GLABRA3 (EGL3) and the WD40-repeat–containing protein TRANSPARENT TESTA GLABRA (TTG). These proteins can bind to the MYB proteins WEREWOLF (WER) and CAPRICE (CPC) to form two protein complexes (the WER- and CPC-complexes, respectively).

Genetic and biochemical studies have highlighted a number of basic features of the epidermal interaction network. First, the WER-complex represses *GL3/EGL3* transcription and enhances *CPC* transcription [6–8]. The CPC-complex is believed to lack transcriptional activity, but CPC has been reported to repress *WER* transcription [8]. Second, the CPC and GL3 proteins exhibit striking mobility, moving freely between epidermal cells [9–11]. Third, the SCRAMBLED (SCM) receptor-like kinase is believed to play a role in the interpretation of a cortical signal that biases pattern formation by repressing *WER* transcription in the H position [12,13]. These network features have been proposed to underlie a pattern-forming mechanism based on lateral inhibition [8], but a detailed investigation of their sufficiency to account for experimental data has not been carried out. It has been suggested on theoretical grounds, however, that autoregulation of WER activity is necessary for epidermal pattern formation [14,15], although experimental support for this proposal is lacking [14]. In this paper, we show by a combination of mathematical modelling and experimental studies that WER autoregulation does not play a significant role in the epidermal patterning network, and propose a mechanism for patterning that depends on the mutual support of the two epidermal cell fates.

## Results

### Mathematical Representation of the Epidermal Patterning Network

**Figure 2:**
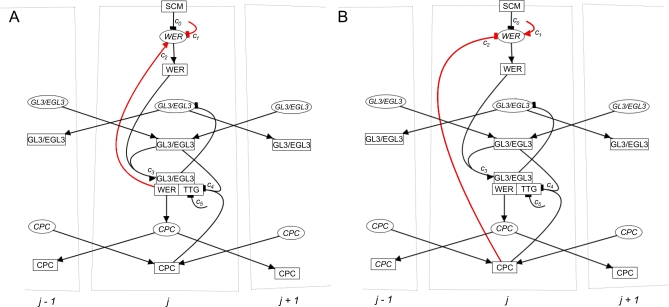
Schematics Showing Two Alternative Forms of the Epidermal Interaction Network (Cell-Net) Based on Known Interactions and Protein Mobility Cell-nets are labelled *j*−1, *j*, *j*+1 according to their position in an epidermal ring (epi-net). mRNAs are represented by ellipses, and proteins by rectangles. All components of a cell-net are shown in cell-net *j*, but for clarity, only mobile proteins (and their corresponding mRNAs) are shown in cell-nets *j*−1 and *j*+1. Arrows between components represent regulatory interactions, with pointed and blunt ends representing activation and repression, respectively. The interactions that differ between the two networks are shown in red. The parameters *c_i_* determine the relative strengths of key interactions, and are shown alongside the arrows representing these interactions. (A) The local *WER* self-activation model. (B) The mutual support model.

**Figure 3:**
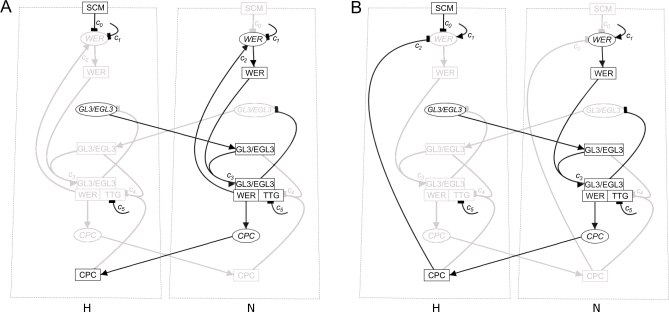
Schematics Showing, in Black, the Expected Component Expression and Active Interactions for Cell-Nets Corresponding to Trichoblast Cells (in the H Position) and Atrichoblast Cells (in the N Position) for the Two Alternative Mechanisms in a Wild-Type Simulation (A) The local *WER* self-activation model. (B) The mutual support model.

We have developed a mathematical model representing the core epidermal interaction network, in order to investigate the regulatory logic of epidermal patterning. Our model seeks to capture all key interactions and protein movements identified in experimental studies (Figure 2). The nature of the regulation of *WER* transcription is central to our model. *WER* transcription is repressed by both SCM and CPC, but no specific activators of *WER* transcription have been identified. To address directly the open question of the necessity for WER autoregulation, we consider two alternative forms of *WER* regulation. In the first version, we assume local *WER* self-activation, implemented by the enhancement of *WER* transcription by WER-complex (Figures 2A and 3A). In this scenario, CPC down-regulates *WER* indirectly via competition for TTG/GL3/EGL3. In the second version, we do not include local *WER* self-activation, assuming instead that *WER* transcription is activated uniformly in all epidermal cells, with both CPC and SCM (in the H-position cells) repressing *WER* transcription directly (Figures 2B and 3B). We refer to the genetic regulatory network containing the first version of *WER* regulation as the “local WER self-activation model,” and the regulatory network containing the second version as the “mutual support model.”

In order to focus more clearly on the core logic of the epidermal patterning network, the model incorporates a number of simplifying assumptions. First, since the expression pattern of TTG within the epidermis is not known, we assume that it is expressed uniformly and that it plays only a permissive role in allowing the formation of WER and CPC protein complexes with GL3/EGL3. On the basis of this assumption, we do not include an explicit TTG variable in our mathematical model. TTG is, however, present implicitly in all the cells of our model epidermis. Second, we do not include the CPC-complex explicitly in the model; rather, we represent the ability of CPC to compete with WER for binding to TTG/GL3/EGL3 [16] by a direct inhibition of WER-complex formation by CPC. The CPC-complex is implicitly present in all model cells that express both CPC and GL3/EGL3. Third, GL3 and EGL3, which act in a partially redundant manner [11], are represented by a single network component. Similarly, the three MYB proteins CPC, TRIPTYCHON (TRY), and ENHANCER OF TRY AND CPC1 (ETC1), which act in a partially redundant manner [17], are also represented by a single network component (denoted by CPC).

In order to incorporate the observed intercellular movement of the CPC and GL3/EGL3 proteins, we have imposed a specific mechanism in our model: both CPC and GL3/EGL3 proteins are moved actively out of the cells in which they are produced (translated). We adopt this active mechanism to reflect the observed accumulation of these proteins in the nuclei of cells *neighbouring* the cells in which they are produced. A GL3-YFP fusion protein, expressed under the *GL3* promoter in a *gl3* mutant background, accumulates in the nuclei of N-position cells, even though the corresponding mRNA is restricted to H-position cells [11]. Similarly, a HA-tagged CPC protein, expressed under the *CPC* promoter in a *cpc* mutant background, accumulates in the nuclei of H cells, even though its mRNA is restricted to N cells [10]. A CPC-GFP fusion protein can be observed in the nuclei of both cell types [9,10]. However, this protein is expressed at much higher levels than the endogenous CPC protein (due perhaps to protein stabilisation) and causes numerous cells in the N position to adopt the trichoblast fate [10]. These experimental results demonstrate that both CPC and GL3 proteins move away from their sites of production, but the mechanism by which they do this is not known. Given this uncertainty, we have incorporated in our model a simple movement scheme that captures the observed complementary patterns of protein production and accumulation. Possible molecular mechanisms underlying this scheme are discussed below.

We simulate a ring of 16 epidermal cells (which we refer to as the epi-net) following its emergence from the meristem. This represents the stereotypical number of cells found in each epidermal ring in the apical region of the seedling root in which patterning takes place [1,2]. As the cells age (and so move further away from the root apex), occasional anticlinal cell divisions can occur, increasing the number of cells in an epidermal ring [4,5] (the cross-section in Figure 1 shows an example of an older epidermal ring in which this has occurred). However, since we are here modelling the earliest stages of patterning in the epidermis, we do not consider these later events explicitly. Each simulated cell (referred to as a cell-net) contains all the components of the *Arabidopsis* root hair patterning network shown in Figure 2, and so in a simulated cell, any combination of components can be expressed, including the combinations specific to trichoblast or atrichoblast cells. Figure 3 shows the network state (expression of network components and active interactions) in cell-nets corresponding to epidermal cells that have adopted either a stable trichoblast or atrichoblast fate. The mechanistic differences between the local *WER* self-activation and mutual support models are clearly visible in Figure 3.

Since mechanistic details (such as rate laws and the corresponding kinetic parameters) of the epidermal interaction network are not known, a model based on differential equations would involve a large number of unknown parameters. Instead, we adopt a modelling framework that encodes the logical form of interactions. At a given time, the components of a cell-net are either expressed or not. Components that have only positive regulatory inputs (WER, GL3/EGL3, *CPC*, CPC, *GL2*, and GL2—see Figure 2) are expressed if their direct positive regulators are expressed. For example, if *WER* (mRNA) is expressed in a cell-net, then WER (protein) will be expressed. *GL3/EGL3* has one negative input (the WER-complex) and is expressed if its input is not. To specify similar logical rules for the expression of the two components (*WER* and WER-complex) whose production is regulated by a combination of positive and negative regulators would involve making arbitrary assumptions about the dominance of activators or repressors (see Protocol S1). To avoid this, and to allow scope for investigating the effects of any assumptions we make about dominance, we adopt a stochastic formalism in which these components each have a time-evolving probability of expression. The probability of a component being expressed corresponds to the average abundance of that component in the cell. In our formalism, the change in probability over time is determined by the expression of the component's direct regulators and the corresponding activation/inhibition “rates” (which encode the relative strengths of the regulatory interactions). For example, the probability of the WER-complex being expressed is increased by a small amount if both GL3/EGL3 and WER are expressed, and decreased by a small amount if both GL3/EGL3 and CPC are expressed. The incorporation of stochasticity in our model not only increases the investigative scope, but also supplies a form of noise, which is an inherent feature of biological systems and is an integral part of cell differentiation. Furthermore, this stochasticity plays an important role in triggering fate assignment in our model of the *scm* mutant, which lacks positional cues from the cortical cells (see Protocol S1). However, the formalism that we adopt is not intended to provide a detailed representation of the stochastic nature of molecular dynamics in a cell. A detailed description of the modelling formalism and equations can be found in Materials and Methods.

Our stochastic Boolean formalism provides a versatile setting in which to investigate the effects of the relative strengths of combinatorial regulators for a specified regulatory logic. However, the results that we obtain from the model are not dependent on the use of this specific formalism. In particular, the behaviour of the model epidermis can be produced using Boolean models with appropriately chosen deterministic logical functions. In this case, the stochasticity needed to trigger patterning in the *scm* mutant epidermis can be introduced by adopting an asynchronous update scheme (see Protocol S1).

### Simulation of Wild-Type and Mutant Epidermal Cell Fate Patterning

**Figure 4:**
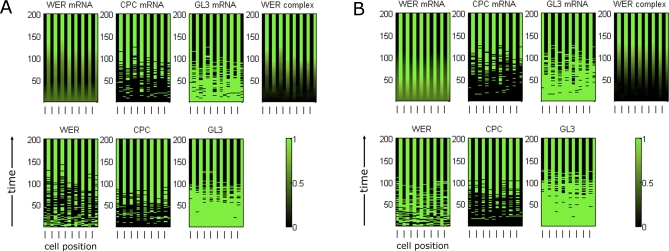
Time-Course Expression Patterns of Network Components in a Simulated Wild-Type Ring of Epidermal Cells Time (in arbitrary units) is displayed on the vertical axis and cell position in the epidermal ring along the horizontal axis (the cells at the left- and right-hand ends are actually neighbours in the epidermal ring). For the two network components whose expression is probabilistic (*WER* mRNA and the WER-complex), the graphs show the probability of expression ranging from 0 (black) to 1 (green). All other components are either expressed (1) or not (0) at any given time. Odd-numbered cell-nets are in the H position, indicated by a dash on the bottom horizontal axis; even-numbered cell-nets are in the N position. Only CPC and *GL3/EGL3* are expressed in cell-nets in the H position, in accordance with experimental data. (A) The local *WER* self-activation model, Figures 2A and 3A; Equation 1. (B) The mutual support model, Figures 2B and 3B; Equation 2.

To assess the ability of the model networks to account for observed wild-type expression patterns, we simulated epi-nets in which all network components (except SCM) were initially expressed at the same level in all cells (i.e., all cell-nets are initially identical). To represent the positional bias received from the underlying cortex, SCM was set to be active only in cells located in the H position, resulting in a lower transcription rate of *WER* than in the N position. In an epi-net, H and N positions alternate: odd-numbered cell-nets are in the H position, while even-numbered cell-nets are in the N position (see Figure 4). With this imposed positional bias, both the local *WER* self-activation and mutual support models are capable of generating stable expression patterns that agree with the expression patterns observed in experimental data (Figure 4).

**Figure 5:**
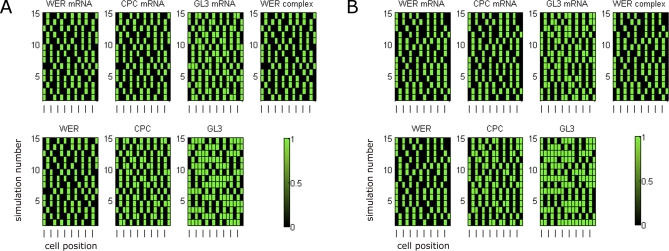
Simulated Expression Patterns in a *scm* Mutant Epidermis The graphs show composites of the steady states resulting from 15 independent simulations in rings of cell-nets, aligned vertically to produce a virtual epidermis. Note that the layers in the simulations are independent (no “longitudinal” signalling or cell aging is included). As observed experimentally, each cell-net adopts a coherent set of expression levels, corresponding to either the trichoblast or atrichoblast fate, but the positions of the two cell fates are not strictly correlated with the H and N positions [12,13]. (A) The local *WER* self-activation model. (B) The mutual support model.

In *scm* mutant plants, experimental data show that epidermal cells adopt well-defined fates, but in a pattern that is not strictly correlated with position relative to the cortex [12,13]. To assess whether the model networks can also account for this phenotype, we set SCM to be inactive in all cells. In these simulations, the only patterning cues come from the stochasticity inherent in our modelling approach (we do not incorporate stochasticity in the initial conditions). Figure 5 shows a composite of the steady states resulting from 15 independent simulations in rings of cell-nets, aligned vertically to produce a virtual epidermis. However, it is important to note that such a picture does not represent the result of a full two-dimensional simulation, including aging and longitudinal signalling between cell rings. Both the local *WER* self-activation and mutual support models develop stable patterns in which each cell-net adopts a coherent state (either trichoblast or atrichoblast). For both models, the patterns produced are qualitatively comparable to those observed in *scm* mutant roots [12,13]. The total removal of cortical bias in our simulations may not be entirely equivalent to the situation pertaining in *scm* mutant roots, as the phenotypes of existing *scm* alleles suggest that some cortical positional information persists in these cases [13]. However, our simulations show clearly that both forms of the epidermal patterning network are capable of *spontaneous* pattern formation, even in the absence of spatial bias. For both local *WER* self-activation and mutual support, the stochasticity in our modelling formalism acts to break symmetry allowing a spatially patterned state to emerge from a spatially uniform initial state.

**Figure 6:**
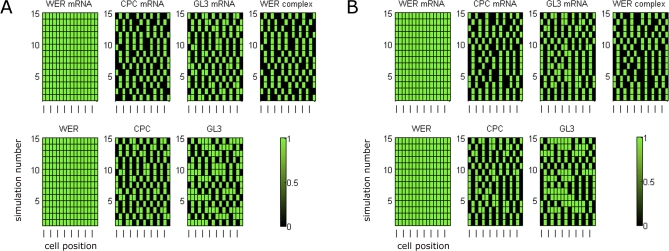
Simulated Expression Patterns in a *WER* Overexpression Epidermis As in Figure 5, the expression patterns for 15 independent simulations are shown. (A) The local *WER* self-activation model. (B) The mutual support model. For both models, the expression of components other than *WER* mRNA and WER protein are as in the *scm* mutant (Figure 5), mirroring experimental observations [8,14].

To simulate the effect of other mutations, we set the corresponding cell-net components to be inactive in all cell-nets. Simulations of a *wer* mutation (unpublished data) result in identical expression patterns for both models, in agreement with experimental data (namely, the uniform expression of GL3/EGL3) [8,11,17]. We simulate *WER* overexpression by imposing uniform expression of both *WER* mRNA and WER protein throughout the epi-net. The epi-net steady states resulting from 15 independent simulations of the two versions of the epidermal patterning networks are shown in Figure 6. The expression pattern of all network components other than *WER* mRNA and WER are as in the simulated *scm* mutant (Figure 5), with each cell-net adopting a coherent state corresponding to either a trichoblast or atrichoblast. This mirrors the expression patterns reported in [8,14] and reflects the fact that *WER*, when overexpressed uniformly, is no longer able to respond to an imposed cortical bias.

### WEREWOLF Does Not Autoregulate during Epidermal Cell Fate Assignment

**Figure 7:**
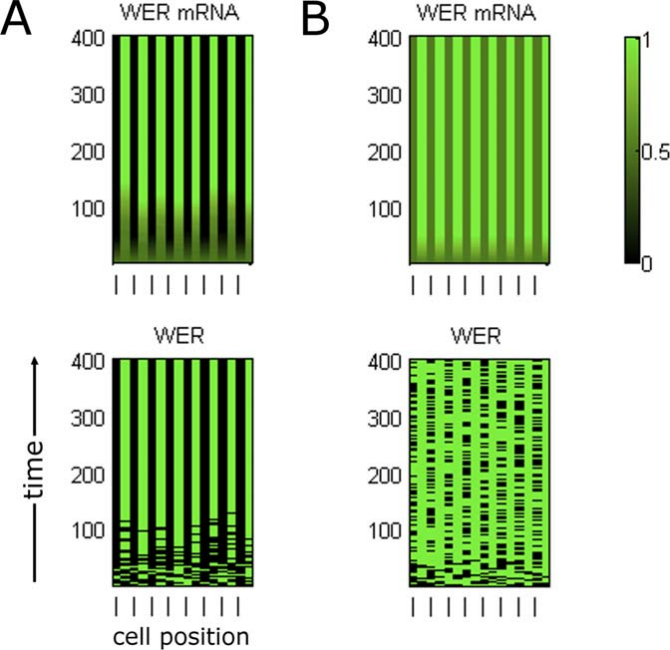
Time-Course Expression Patterns of WER mRNA and Protein in a Simulated *cpc* Mutant Other network components (not shown) have identical expression for both mechanisms. (A) In the local *WER* self-activation model (Figures 2A and 3A), the local activation of *WER* expression by the WER-complex results in a wild-type pattern of *WER* expression even in the absence of CPC. (B) In the mutual support model of Figures 2B and 3B, the loss of CPC-mediated repression of *WER* results in an increase in WER expression in the H positions because it is only being repressed by SCM. This is also observed experimentally [8].

Figure 7 shows the expression of *WER* mRNA and WER protein in a simulated *cpc* mutant. Although both the local *WER* self-activation and mutual support models generate expression patterns for most network components that are in line with experimental data [11], they generate significantly different patterns of *WER* expression. In the local *WER* self-activation model (Figure 2A), the activation of *WER* expression by the WER-complex results in a wild-type pattern of *WER* expression even in the absence of CPC (Figure 7A, cf. Figure 4A). In contrast, the loss of CPC-mediated repression of *WER* in the mutual support model (Figure 2B) results in an increase in *WER* expression in the H positions, as it is only being repressed by SCM in the absence of CPC (Figure 7B). This corresponds to the pattern of *WER* expression observed experimentally [8]. This result suggests that the mutual support model, which does not incorporate local *WER* self-activation, more accurately reflects events occurring during the patterning of the epidermis.

**Figure 8:**
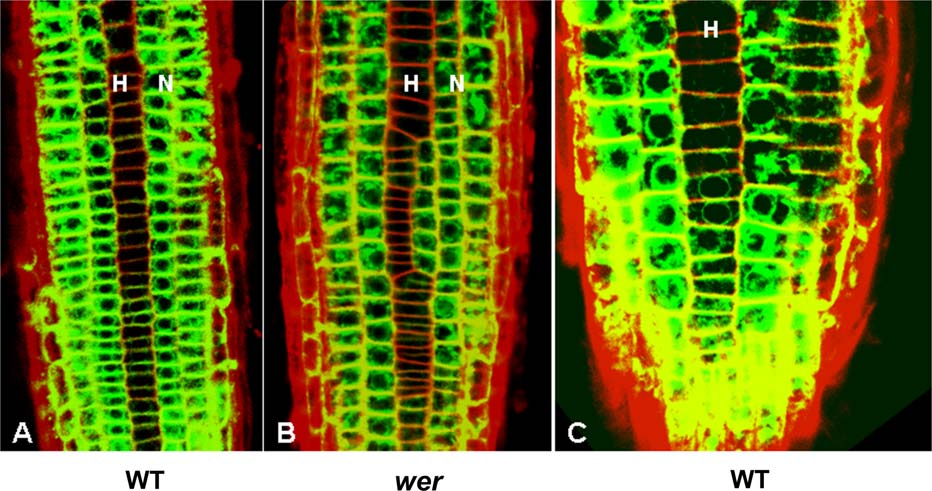
*WER* Transcription Is Not Autoregulated and Initially Occurs in All Root Epidermal Cells before Being Restricted to Predominantly N Cell–Specific Activity (A and B) *WERpro::GFP* activity in wild-type (A) and *wer* mutant (B) backgrounds. The preferential activity of the *WER* promoter in N-position cell files (marked N) as opposed to H-position cell files (marked H) is maintained in the *wer* background. WT, wild type. (C) *WERpro::GFP* activity in a H cell file in a wild-type root meristem. Near the apex of the root (bottom of the image), *WER* promoter activity is uniformly high throughout the epidermal rings. *WER* promoter activity gradually decreases in H position cells as they move away from the meristem.

Since the local *WER* self-activation model fails to reproduce the observed pattern of *WER* expression in the *cpc* mutant, we tested the ability of the WER-complex (or WER) to enhance *WER* expression by examining the expression of GFP driven by the *WER* promoter (*WERpro::GFP*) in a *wer* mutant background (using a null mutant in which no functional WER protein is produced). We found GFP expression to be the same in wild type and the *wer* mutant, showing that *WER* transcription does not depend on the presence of functional WER protein (Figure 8A and 8B). To test directly our alternative assumption that *WER* transcription is activated uniformly in all epidermal cells, we carefully examined *WER* promoter activity (as visualised by *WERpro::GFP*) in wild-type seedlings. Whereas *WERpro::GFP* is preferentially expressed in the N cell file in less apical cells of the meristem, it exhibits uniform activity between N and H cell positions in cells proximal to the initials (Figure 8C). These results show that the initially uniform activity of the *WER* promoter throughout the epidermis resolves rapidly into a pattern matching that of *WER* transcription in wild-type roots even in the absence of WER protein. This strongly suggests that the establishment of patterned *WER* transcription—a key event in epidermal patterning—does not depend on local WER self-activation. Since the pattern of *WER* promoter activity in both wild-type and *wer* mutant roots corresponds to the wild-type pattern of cell fate in the epidermis, there is no obvious role for posttranscriptional regulation of WER activity (since posttranscriptional regulation of WER can only occur in cells in which *WER* is transcribed). Taken together, our modelling and experimental results show that *WER* is initially activated uniformly in the epidermis, and suggest that its rapid repression in emerging trichoblasts is controlled by a combination of SCM-mediated positional information and CPC.

**Figure 9:**
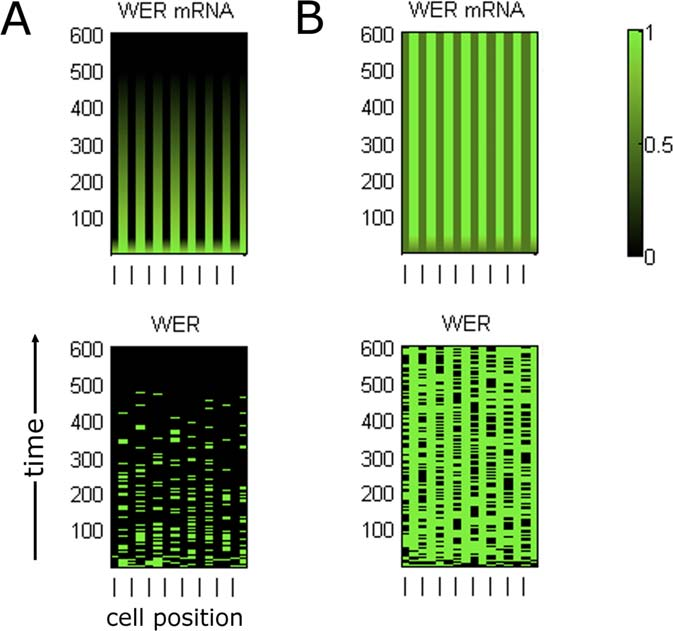
Time-Course Expression Patterns of *WER* mRNA and WER Protein in a Simulated *gl3 egl3* Double-Mutant Ring of Epidermal Cells Other network components (not shown) have identical expression patterns for both mechanisms. (A) Local *WER* self-activation model (Figures 2A and 3A): Since *WER* is no longer up-regulated by the WER-complex (as the WER-complex cannot form in a *gl3 egl3* double mutant), the probability of *WER* expression reduces to zero in all cells. (B) Mutual support model (Figures 2B and 3B): In this mechanism, *WER* is up-regulated uniformly in all cell-nets, and down-regulated by CPC and SCM in cells in the H positions. However, as there is no WER-complex, there is no CPC, and so *WER* is down-regulated by SCM alone, resulting in patterned *WER* expression with an increased probability of expression in the H positions (compared to wild type). The magnitude of this increase depends on the relative strengths of *WER* down-regulation by CPC and SCM. This pattern mirrors the pattern of expression of GFP driven by the *WER* promoter shown in Figure 10.

**Figure 10:**
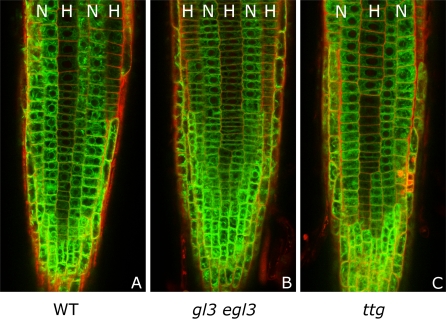
*WERpro::GFP* Activity in Wild-Type (A), *gl3 egl3* Double-Mutant (B), and *ttg* Mutant (C) Backgrounds The preferential activity of the *WER* promoter in N-position cell files (marked N) as opposed to H-position cell files (marked H) is maintained in both mutant backgrounds. No reduction in the level of promoter activity is observed in the mutant lines. WT, wild type.

To explore further the differences between the two model networks, we simulated mutants that are incapable of forming the WER-complex. Since GL3/EGL3 and TTG are required for complex formation, both the *gl3 egl3* double mutant and the *ttg* mutant should lack WER-complex. In this scenario, the local *WER* self-activation and mutual support models predict different patterns of *WER* expression. In the local *WER* self-activation model, the failure of WER-complex formation results in a uniform loss of *WER* expression in the model epidermis (Figure 9A). However, since *WER* expression does not depend on local self-activation in the mutual support model, *WER* is expressed in an essentially wild-type pattern in the model epidermis (with an increased probability of expression in cells in the H position due to the lack of CPC-mediated repression) (Figure 9B). To test this prediction experimentally, we examined the expression of GFP driven by the *WER* promoter (*WERpro::GFP*) in these mutant backgrounds. As predicted by the mutual support model, GFP expression is essentially the same in the wild-type and mutant epidermis (Figure 10). This supports our finding that WER self-activation does not play a significant role in the early stages of epidermal patterning, and provides direct experimental validation of the predictions of the mutual support model of epidermal patterning.

## Discussion

Taken together, our modelling and experimental studies support a mechanism for spatial pattern formation in the *Arabidopsis* root epidermis that depends critically on the movement of mobile proteins between cells—a lateral inhibition with feedback (LIF) mechanism. Importantly, this mechanism does not depend on local *WER* self-activation, but relies instead on the repression of *WER* transcription in emerging trichoblasts by CPC protein. Previous theoretical discussions of epidermal patterning [14,15] have suggested that local *WER* self-activation is a necessary feature of the patterning network—a local activation and lateral inhibition (LALI) mechanism [18,19].

Although both the LALI and LIF mechanisms can generate similar stable patterns of cell fate, the logical structure of the underlying networks is quite different. LALI mechanisms depend on interlinked positive feedback (short range) and negative feedback (long range) whereas LIF depends on a single “double-negative” feedback loop, mediated by intercellular signalling, and does not depend on local self-activation. The logical structure of the LIF mechanism is analogous to the Delta-Notch signalling system in animal epithelia, in which proneural activity in one cell represses proneural activity in its neighbours through the transmembrane ligand Delta and its receptor Notch, ensuring directional signalling. Models of the Delta-Notch system exhibit spontaneous patterning that does not depend on any local self-activation [20,21].

In the LALI mechanism, the “activated” cell state (atrichoblast) inhibits its neighbours, which adopt an alternative fate (trichoblast). In contrast, in the LIF mechanism, cells adopting one of the two epidermal fates are mutually supporting, producing factors required by cells adopting the alternative fate. Adoption of the atrichoblast fate (high WER-complex) requires GL3/EGL3 from neighbouring cells; adoption of the trichoblast fate (low WER-complex) requires CPC from neighbouring cells (to prevent accumulation of WER-complex). In other words, a cell can only have high levels of WER-complex if a neighbouring cell has a low level of WER-complex and vice versa. This model therefore predicts that “runs” of three or more epidermal cells with similar levels of WER-complex should not occur. In the root apical meristem, where the early patterning of gene expression in the epidermis occurs, each ring of epidermal cells contains 16 cells, with alternating cells in H and N positions, as encoded in our model [22]. We therefore observe a strict alternating pattern in our wild-type simulations that incorporate a positional bias from the cortex. In the simulated *scm* mutant, which lacks cortical bias, we do not observe more than two cells of the same fate neighbouring each other. In growing roots, the number of cells in an epidermal ring tends to increase as cells move away from the root apical meristem, due to occasional anticlinal cell divisions [4,5]. This is shown clearly in Figure 1, in which most H-position cells are separated by two N-position cells. In older epidermal rings, three or more adjacent cells are sometimes observed to have the same pattern of gene expression, which cannot be accounted for by our early patterning network in its current form. However, it is likely that once the basic pattern of expression of the core epidermal patterning components has been established, cell fate is stabilised by additional factors such as chromatin modification [23–25]. Such fate stabilisation mechanisms would allow cells to maintain their network state even when no longer supported by a neighbouring cell of the alternate fate.

We have shown by model simulation that the LALI mechanism (incorporating local *WER* self-activation) fails to account fully for the previously reported phenotype of a *cpc* mutant root, and by experiment that a specific form of local self-activation (WER-mediated up-regulation of *WER* transcription) does not operate in the early patterning of the root epidermis. Our combined modelling and experimental results favour an alternative mechanism (LIF) in which the two emerging cell fates mutually support each other through the active exchange of the mobile proteins CPC and GL3. The mutual support model predicts patterns of WER promoter activity in *wer*, *gl3 egl3*, and *ttg* mutant roots that are similar to wild type. We have verified these predictions experimentally, providing validation for the model and further support for our proposed patterning mechanism. Importantly, the model based on the LALI mechanism does not account for these new observations.

The mutual support model incorporates the active movement of the CPC and GL3 proteins from the cells in which they are produced to neighbouring cells. Such an active mechanism is suggested by the previously reported complementary patterns of production and accumulation of these proteins in the epidermis. We have adopted a modelling formalism based on binary states of expression (“on” or “off”). In this formalism, the patterning of the model epidermis depends on this active mechanism of protein movement. However, the possibility remains that the observed complementary patterns of protein production and accumulation could result from simple diffusion of the proteins between cells, together with sequestration of the proteins into nuclear-localised protein complexes (as occurs in the directed movement of the SHORTROOT protein in the root apical meristem [26]).

Previous theoretical discussions of epidermal patterning have proposed that local self-activation is a necessary feature of a patterning mechanism [14,15]. This conclusion is based on the theory of two-component activator–inhibitor models in which movement is purely diffusive. To explore the validity of this conclusion for the root epidermal patterning network, we have analysed two different mathematical representations of the mutual support model. First, we have developed a logical state (Boolean) model in which CPC and GL3 protein movement depends on a movement parameter, allowing both active and passive (diffusion-like) movement to be represented. Analysis of this model shows that passive GL3 movement is sufficient to account for patterning, so long as CPC moves actively (see Protocol S1). Second, we have developed a reaction–diffusion analogue of our logical model in which both GL3 and CPC move between cells by simple diffusion alone (see Protocol S1). When reduced to an effective two-component model for GL3 and CPC (by assuming that protein complex formation and WER dynamics reach equilibrium much faster than diffusive processes), we show that the model can take the form of a cross activator–inhibitor system, which is capable of spontaneous pattern formation via diffusion-driven instability [27]. This analysis shows that the mutual support mechanism we propose can generate pattern spontaneously by diffusive protein movement and protein complex formation, in the absence of any local self-activation reaction. Numerical simulation of both the full and reduced models confirms that the diffusive mechanism generates stable patterns with protein distributions that match those observed in the root epidermis (see Protocol S1).

Our results serve to highlight the importance of a detailed investigation of the mechanisms of the intercellular movement of proteins such as CPC and GL3/EGL3 [28]. A number of simple mechanisms might underlie an effective directionality of protein movement away from producing cells. For example, the movement of proteins through plasmodesmata could be dependent on a chaperone protein that is produced only in cells producing the mobile protein. Alternatively, passage through plasmodesmata could depend on localisation of the protein in the endoplasmic reticulum, which would favour movement away from the cells in which the protein is translated. An intriguing parallel is provided by the movement of small metabolites through small intercellular pores (microplasmodesmata) in the filamentous cyanobactoria *Anabaena*. A recent study has shown that the permeability of pores (and hence the mobility of metabolites) mirrors the states of differentiation of the two cell types in this system [29]. In particular, as individual cells in the filament move towards a differentiated heterocyst fate, the permeability of pores between emerging heterocysts and neighbouring vegetative cells decreases compared to that between two vegetative cells. Thus, in this very different system, differential permeability of intercellular channels, dependent on cell fate, can establish spatially patterned protein distributions. The widespread occurrence of cell-to-cell trafficking of macromolecules in plant and animal tissues [30] suggests that mechanisms of the type we describe—centred on mobile proteins that can be sequestered in protein complexes—may play a role in a range of pattern-forming processes operating in planar groups of cells.

## Materials and Methods

### *WERpro::GFP* analysis in mutant roots.

The *WERpro::GFP* construct was previously reported in [31]. Briefly, it included a 2.5-kb *WER* promoter fragment 5′ to the GFP coding sequence and a 1.1-kb 3′ *WER* fragment, and faithfully reported the *WER* transcription pattern. To examine the expression of *WERpro::GFP* in the *wer*, *gl3 egl3*, and *ttg* mutant backgrounds, we used the published *wer* allele, *wer-1* [31], the *gl3–1 egl3–1* line [11], and the *ttg1–13* mutant [2]. Plants homozygous for the *WERpro::GFP* insertion were crossed to plants homozygous for one of the mutant alleles. The resulting plants were self-pollinated, and F2 plants that were homozygous for the *wer-1*, *gl3–1 egl3–1*, *or ttg1–12* mutations and the *WERpro::GFP* transgene were selected. These plants were in turn self-pollinated to produce a population of seed that were homozygous for the desired mutant allele and the *WERpro::GFP* transgene.

For confocal microscopy imaging, 4- or 5-d-old roots were stained with 10 μg/ml propidium iodide and visualised on a Leica TC5 SP confocal microscope. Images were assembled using Adobe Photoshop.

### Mathematical formalism—epidermal patterning network.

In our models, a ring of 16 epidermal cells (the stereotypical number found in the apical region of the seedling root in which patterning takes place) is represented by an epi-net comprising 16 identically composed cell-nets, indexed by the integer *j* = 1, 2, …, 16. The set of components in each cell-net, together with their interactions, is shown schematically in Figure 2. In the mathematical model, the state of mRNAs is represented by the corresponding gene name abbreviation (for example, *CPC^t^_j_* represents the state of *CPC* mRNA at time *t* in cell-net *j*). The state of the corresponding protein carries an appended “p” (for example, WERp*^t^_j_* represents the state of WER protein at time *t* in cell-net *j*). The state of the WER-complex is denoted by WERc. In order to capture what we believe to be the essential logic of the epidermal patterning network, while keeping the number of distinct molecular species in the model to a minimum, a number of known network components have been left out of the model, or combined into a single model variable. Both GL3 and EGL3 are represented jointly by a single model element GL3 (comprising variables for mRNA and protein). We justify this simplification by noting that all published data suggest that GL3 and EGL3 are regulated similarly and exhibit functional redundancy. Similarly, we represent the three single-repeat R3 MYB proteins CPC, TRIPTYCHON (TRY), and ENHANCER OF TRY AND CPC1 (ETC1) by a single model element CPC, since experimental evidence supports the idea that they act collectively and redundantly to specify the trichoblast fate [17]. Furthermore, in the absence of experimental data to the contrary, we assume that the WD-repeat protein TRANSPARENT TESTA GLABRA (TTG), an essential component of the WER-complex, is expressed uniformly throughout the epidermis. This assumption renders the explicit representation of TTG in the models unnecessary, and our models do not contain TTG variables (although the protein is implicitly assumed to be present in all cells).

To investigate the patterning potential of the local *WER* self-activation and mutual support models (Figure 2), we use a discrete-time logical formalism. In this approach, the state of each network component is represented by a binary variable taking either value 1 (component expressed) or 0 (not expressed). Time evolution of the network state is modelled by the synchronous update of the state of each network component at equally spaced time points (*t*, *t*+1, *t*+2, …). For the network components whose state is regulated by only one other component type (WERp, *GL3*, GL3p, *CPC*, or CPCp), we adopt a conventional deterministic Boolean update formalism [32]. For the two components whose state is regulated by more than one input (*WER* and WERc), we adopt a novel formalism based on the *probability P^t^_j_*[X] that the state of component *X^t^_j_* will be 1 at time *t*. Rather than specifying a deterministic function for the time evolution of the states of these components, we instead specify a deterministic rule for the time evolution of the probability of expression. This form of update allows us both to vary the relative strengths of the inputs and to incorporate stochasticity in the update process. Although this approach directly introduces stochasticity into the evolution equations of only two network variables, the stochasticity filters through to the other components. Thus, whereas all components could be represented probabilistically, this would necessitate the introduction of many more undetermined parameters without adding further functionality to the model. In a simulation of the network, the actual values (0 or 1) of *WER^t^_j_* and WERc*^t^_j_* are determined stochastically at each time step according to the probability of expression, *P^ t^_j_*[*WER*] and *P ^t^_j_*[WERc].

The parameters in our probabilistic update functions (see below) allow us to explore the robustness of patterning to changes in the relative strengths of the inputs. Furthermore, the incorporation of stochasticity into the system is important, since stochasticity is an inherent feature of biological networks and is required in our models to initiate patterning in the simulated *scm* mutant. However, our approach does not attempt to mimic any specific form of stochasticity found in biological systems, and we have shown that the results obtained using our probabilistic formalism can be reproduced by using a deterministic Boolean model with stochasticity introduced in the form of asynchronous state update (see Protocol S1).

Our probabilistic Boolean formalism provides a simple way of exploring the consequences of specific assumptions about the regulatory logic of the epidermal patterning network. However, the use of a logical (on/off) representation of the network state assumes that the regulatory interactions represented in the model (e.g., transcription and translation) are essentially “all or nothing.” Since our primary objective is to explore the *differences* between two alternative network structures, we believe that this assumption is appropriate. Other approaches to modelling regulatory networks, such as those based on differential equations, do not depend on such an assumption being made. However, these models require the specification of many more parameters than our model, to represent the details of specific interaction kinetics. Such models can provide more-realistic representations of the dynamical evolution of the state of the network. Given that there are currently no data, either from which appropriate parameters can be specified, or against which detailed network dynamics can be validated, we do not believe that these approaches currently have a significant advantage over our logical formalism.

The local *WER* self-activation and mutual support models are defined in Equations (1) and (2), respectively. The models are identical apart from the equation encoding the time-evolution of *WER* mRNA. The symbol ∨ represents the logical “inclusive OR” function (i.e., A∨B = 0 if and only if A = B = 0). *c*_0_, *c*_1_,...*c*_5_, are positive parameters that determine the relative strengths of the inputs in the probabilistic multi-input update functions for *WER* and WERc (Figure 2). The constant terms *c*_1_ and *c*_5_ represent either constitutive production or degradation, depending on their preceding signs. The regulatory inputs to *WER* and WERc specify the amount by which the probability of expression of these components *changes* during a single time step. This form of update rule is similar to the rate equations that form the basis of differential equation models (in which the rate of change of a component is determined by the values of its direct regulators). Values within the brackets ⌊ ⌋ are forced to remain between 0 and 1.


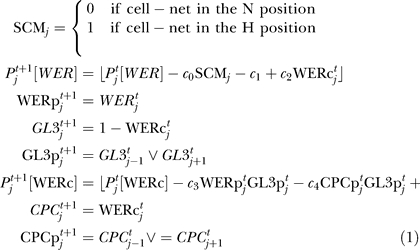



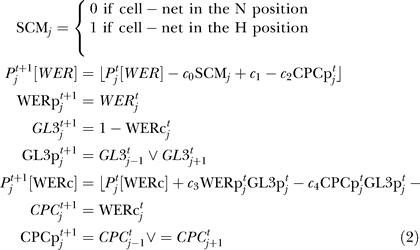


A positional bias from the underlying cortex is incorporated in the models via the state of the SCRAMBLED (SCM) receptor-like kinase, which is taken to be 1 in cell nets occupying the H position and 0 in cell-nets occupying the N position. Activity of SCM results in a reduction in the rate of transcription of *WER*, determined by the parameter *c*_0_. We assume the two positions to be arranged alternately, as is typically the case in the apical root epidermis (anticlinal cell divisions in the epidermis, which can increase the spacing between H-position cells, typically occur further from the meristem, where the expression pattern of network components has already stabilised).

The initial state of all components, bar SCM (see above), is identical in all cell-nets, representing the fact that the final stable state of each cell-net is determined by its position relative to the underlying cortical cells rather than cell lineage. As the state of each cell-net evolves in time, the cell-nets adopt stable patterns of expression corresponding to either the trichoblast or atrichoblast cell fate (Figure 4). A detailed discussion of the dependence of the behaviour of the models on initial conditions and parameter values can be found in Protocol S1.

## Supporting Information

Protocol S1 Protocol S1 Contains Parameter and Initial Condition Analysis of the Mathematical Models, and Details of a Reaction–Diffusion Model for Epidermal Patterning This file includes nine supporting figures and four supporting tables.
(8.35MB DOC).

## Abbreviations

LALI: local activation and lateral inhibition
LIF: lateral inhibition with feedback
