# Supplementary material for: A Mutual Support Mechanism through Intercellular Movement of CAPRICE and GLABRA3 Can Pattern the Arabidopsis Root Epidermis
Source: PLoS Biol. 2008 Sep 23;6(9):e235. doi: 10.1371/journal.pbio.0060235 (PMC2553841; doi:10.1371/journal.pbio.0060235)
Supplement: Protocol S1 — This file includes nine supporting figures and four supporting tables. (8.35MB DOC). [file pbio.0060235.sd001.doc]

**Mutual support of alternative cell fates through intercellular movement of CAPRICE and GLABRA3 provides a mechanism for patterning the *Arabidopsis* root epidermis**

# Supporting Protocol S1

Natasha Saint Savage^1^, Tom Walker^2^, Yana Wieckowski^3^, John Schiefelbein^3^, Liam Dolan^2^, Nicholas A. M. Monk^4,5*^

*^1^* *Department of Molecular Biology and Biotechnology, University of Sheffield, Firth Court, Western Bank, Sheffield, S10 2TN, UK*

*^2^Department of Cell and Developmental Biology, John Innes Centre, Colney, Norwich, NR4 7UH, UK*

*^3^ Department of Molecular, Cell, and Developmental Biology, University of Michigan, 830 North University Avenue, Ann Arbor, MI  48109, USA*

*^4^ Author for correspondence at current address: Division of Applied Mathematics, School of Mathematical Sciences, University of Nottingham, University Park, Nottingham, NG7 2RD, UK*

*^5^ Centre for Plant Integrative Biology, School of Biosciences, University of Nottingham, Sutton Bonington Campus, Loughborough, LE12 5RD, UK*

^*^Corresponding Author

**General bounds on parameter values in the probabilistic Boolean formalism**

Equations (1) and (2) (in the main manuscript) describe reactions within and between cell-nets (the mathematical representation of epidermal cells). The parameters *c_k_* play a central role in the time-evolution of the states of cell-nets. *c_k_* determine how the expression probabilities of *WER* and WER-complex change over time in relation to the expression of their multiple inputs. The *c_k_* must satisfy certain bounds in order for cell-nets to be able to adopt stable states corresponding to either the trichoblast or atrichoblast fate, as dictated by biological data. Satisfaction of these bounds does not guarantee that a spatially alternating pattern of cell fates will always be reached by the model (Table S2).

*Bounds common to both local WER self-activation and mutual support models (Eqs. (1) and (2)):*

**(1) Regulation of WER-complex: *c*_3_ *> c*_5_**

*c*_5_ represents a basal degradation rate and *c*_3_ represents the formation rate of WER-complex. For the WER-complex to be able to have a stable probability of expression greater than zero it is necessary that *c*_3_ *> c*_5_.

**(2) Local WER self-activation: *c*_2_ > *c*_1_**

For the local *WER* self-activation model this bound ensures that if WER-complex is expressed in a cell-net then it increases the probability of *WER* expression. In the mutual support model this bound ensures that if CPC is expressed in a cell-net then it decreases the probability of *WER* expression.

*Bound specific to the local WER self-activation model (eqs. (1)):*

**(3) *WER* initial conditions: $P_{j}^{0}[\text{WER}]$ > 0**

The initial probability of *WER* being expressed must be greater than zero for any patterning to take place, since *WER* self-regulates.

**Parameter values and initial conditions used in simulations**

The following sets of parameter values, which satisfy the bounds specified above, were used for simulation of the wild type epidermis:

*Local WER self-activation (Eqs. (1)):* *c_k_* = {0.01, 0.001, 0.01, 0.02, 0.02, 0.001}

*Mutual support (Eqs. (2)):* *c_k_* = {0.01, 0.01, 0.015, 0.02, 0.02, 0.01}.

The numerical differences between the two parameter sets were chosen so the time taken to reach steady state is roughly equal for both reaction networks.

The appropriate size of a time-step in the model is dependent on the values of the parameters *c_k_*, which encode the ‘response speed’ of the network. In the absence of detailed kinetic data, we assume a time-step to be around 15–30 minutes, corresponding to fast (but still plausible) component response times. The number of time-steps taken to achieve a stable pattern of network activity depends on the values assigned to the *c_k_* (see Table S2). Given a time-step of 15–30 min., the time taken to achieve a stable network state in our wild type simulations is around 34–69 hours.

In our simulations, the states of all model components are taken to be spatially uniform initially. All components except for *WER* are either not expressed or have zero probability of being expressed initially. *WER* has an initial probability of expression of 0.5 in all cell-nets.

# Dependence of model steady states on initial conditions

The epi-net (which represents a ring of *Arabidopsis* root epidermal cells) comprises 16 cell-nets with 8 equally-spaced hair positions. The ‘target’ steady state pattern for simulations of wild type roots is one in which trichoblasts alternate with atrichoblasts (which we refer to as the alternating steady state). The states and probabilities of expression of network components for each cell-net in the alternating steady state are shown in Table S1. Substitution of the values in Table S1 into Equations (1) for the local *WER* self-activation model and Equations (2) for the mutual support model confirms that the alternating steady state is indeed a steady state and that it cannot be left once it is reached. In the following, we consider only the LIF network, since this best represents experimental expression data.

It is the probabilistic components of the epi-net that are of most interest when studying the dynamics of the system, as the other components are deterministic and are either equal to the probabilistic components or their converse (with a delay of one or two iterations). Thus, when looking at how initial conditions affect the final state of the epi-net we need only consider the initial probabilities. In addition, as we are investigating cell fate assignment in the root epidermis, the state of the cell-nets should be initially identical, further reducing the number of initial conditions. We set three distinct initial probabilities for each component (0, 0.5 and 1), resulting in a total of nine distinct initial conditions (shown in Table S3). For each initial condition, 500 wild type simulations were run. The values of *c_i_* were identical to those used for the simulations shown in the main paper (see above). The percentages of simulations entering the alternating steady state for each initial condition are noted in Table S3.

Not all initial conditions guarantee that the system will attain the alternating steady state every time a simulation is performed. The failure of the epi-net to reach the alternating steady state occurs when WER-complex becomes established in the H position; this occurs most frequently for the initial condition *WER* = 1, WERc = 0. In this case, *WER* is initially established in all cell-nets, but the WER-complex has yet to become established and thus early in the simulations there is little to no CPC protein allowing the probability of *WER* remains close to one. As the probability of WER-complex expression starts to increase, the stochastic nature of the dynamics makes it possible for expression of WER-complex to be more frequent in a H position, leading to the inhibition of *WER* (and thus WER-complex) in neighbouring N positions (via CPC). This happens to a lesser extent for initial condition (1,0.5). The same is not true for the initial condition (1,1), in which case WER-complex is fully established and CPC is initially expressed uniformly throughout the epi-net, reducing the probability of *WER* expression from the outset. As this reduction is biased by SCM in the H positions, the alternating steady state is attained.

**Parameter sensitivity**

In this section, we explore the sensitivity of the epi-net’s state to the model parameters *c_k_* for the mutual support model (Eqs. (2)). The simulations of the mutual support model documented in Figs. 4B, 5B, 6B, 7B and 9B were performed using the parameter set *c_k_*  = {0.01, 0.01, 0.015, 0.02, 0.02, 0.01}. To explore parameter space, we varied the value of each *c_i_* from these base values in turn. For each variation, we performed 500 simulations and determined the average number of iterations required for the probabilistic components (*WER* and WER-complex) to reach steady values corresponding to the alternating steady state (Table S1). We focused on the probabilistic components of the network because they dictate the state of their deterministic downstream components. Thus, if the probabilistic components reach the alternating steady state the full network will do so within two iterations. The average numbers of iterations needed to attain steady state are recorded in Table S2 and graphically in Fig. S1, together with the percentages of simulations that attain the alternating steady state. Below, we summarise the dependence of the behaviour of the epi-nets on the model parameters.

***c*_0_ and *c*_1_**

For cell-nets in the N position, the probability of WER expression is increased by *c*_1_ during each time step, while for cell-nets in the H position it is increased by *c*_1_ – *c*_0_ (due to SCM-mediated repression). Thus, altering either of these two parameters alters the positional bias acting on the system. For this reason, the percentage of epi-nets that attain the alternating steady state decreases as either *c*_0_ is decreased or *c*_1_ is increased. In order for epi-nets to be able to attain the alternating steady state, we require 0 < *c*_1_ < 0.025. When *c*_1_ = 0, there is no up-regulation of *WER*, and the epi-net attains a state corresponding to the *wer* mutant expression pattern. When *c*_1_ = 0.025, it is equal to *c*_0_ *+ c*_2_, which is the maximal level of *WER* down-regulation (by SCM and CPC). Consequently, the probability of *WER* expression cannot be reduced and increases to one in all positions, resulting in an expression pattern similar to that obtained in simulations of WER over-expression (Fig. 6).

***c*_2_**

As *c*_2_ is decreased, the number of iterations required to reduce the probability of *WER* expression to zero in the H positions increases. When *c*_2_ = 0 (=*c*_1_ – *c*_0_), the probability of *WER* expression cannot be reduced in the H positions, and the epi-net is unable to reach the alternating steady state. For small values of *c_2_*, as long as *c_2_ > 0* is satisfied the alternating fixed point will eventually be reached. As *c*_2_ is increased, *WER* is more likely to be inhibited (by CPC), leading to an increase in the likelihood of hairs forming in N positions.

***c*_3_, *c*_4_ and *c*_5_**

These parameters control the probability of WER-complex expression. WER-complex is central to the reaction network, since it down-regulates *WER* (in neighbouring cells) via the up-regulation of CPC. Consequently, the numbers of iterations required for WER-complex and *WER* to attain their alternating steady state expression patterns follow a similar trend. We assume that the initial probability of WER-complex expression is zero so the number of iterations required to attain the steady state is determined by the number of iterations required to increase the probability of WER-complex expression to 1 in cell-nets occupying the N position. Thus when the two inhibitory parameters (*c*_4_ and *c*_5_) are increased, the number of iterations required to attain the alternating steady state is increased. The converse holds when we increase *c*_3_. The alternating steady state is reached for all *c*_4_ [0,1], so in this model the epi-net can pattern appropriately without competition between CPC and WER for the TTG/GL3/EGL3 complex. This result is a direct consequence of the assumption that CPC and GL3/EGL3 are actively moved away from the cells in which they are produced.

**Probabilistic expression**

The expression of *WER* mRNA and WER-complex are regulated by both positive and negative regulators. For WER-complex to form, WER must compete with CPC to bind with TTG/GL3/EGL3 and so CPC is a negative regulator of the WER-complex. The regulation of *WER* mRNA is the main focus of our modelling. It is either up-regulated by the WER-complex and down-regulated by SCM (the *WER* self-activation model) or down-regulated by CPC and SCM, and uniformly up-regulated (the mutual support model)—see Fig. 2. Which Boolean functions best describe the above interactions is not clear. Below we show the truth tables for *WER* mRNA and WER-complex in the mutual support model. Both have one ambiguous entry. For the purpose of this discussion we will only use the mutual support model, since it better describes the biological data.

| CPC | SCM | *WER* |
| --- | --- | --- |
| 0 | 0 | 1 |
| 0 | 1 | ***a*** |
| 1 | 0 | 0 |
| 1 | 1 | 0 |

*WER* mRNA

| GL3 | WER | CPC | WERc |
| --- | --- | --- | --- |
| 0 | 0 | 0 | 0 |
| 0 | 0 | 1 | 0 |
| 0 | 1 | 0 | 0 |
| 0 | 1 | 1 | 0 |
| 1 | 0 | 0 | 0 |
| 1 | 0 | 1 | 1 |
| 1 | 1 | 0 | 0 |
| 1 | 1 | 1 | ***b*** |

WER-complex

*WER mRNA:*

As no components of the known network up-regulate *WER* mRNA in the mutual support model we assume uniform up-regulation (due to basal transcription). This can be seen in the *WER* truth table on the top line: if CPC and SCM = 0 then *WER* = 1. If CPC is present in a cell-net then *WER* mRNA is not expressed (biological data suggest that CPC down-regulation is stronger than the uniform *WER* up-regulation). If CPC is not present in the H position (CPC=0, SCM = 1) then, if *a* = 0 (see *WER* mRNA truth table) we are assuming that the cortical bias is stronger than uniform *WER* up-regulation. If *a* = 1 then the cortical bias is not strong enough to overcome uniform *WER* up-regulation. Furthermore, if *a* = 1 the expression of *WER* is independent of SCM (*WER* = 1 – CPC). Biological data suggest that *WER* expression is not independent of SCM, so we have to set *a* = 0.

Returning to the probabilistic equations (2), the equation for *WER* expression is, . When $\text{CPC}_{j}^{t}=0$ and $\text{SCM}_{j}=1$, . Setting *a* = 0 in the truth table is equivalent to setting $c_{0}>c_{1}$ in the probabilistic equation, ensuring (for $\text{CPC}_{j}^{t}=0$ and $\text{SCM}_{j}=1$). From Table S2 we see this is an unnecessarily strong condition, since some values of $c_{0}<c_{1}$ guarantee that the epi-net reaches the alternating fixed point. In addition, wild type experimental data show that trichoblasts occasionally form in the N position, so it may be preferable to vary the relationship between *c_0_* and *c_1_* to better fit this characteristic of the experimental data.

*WER-complex:*

The Boolean function for WER-complex has an ambiguity when GL3, WER and CPC all have value one in a cell-net. If we let *b* = 0 this suggests that CPC has a better binding affinity for TTG/GL3/EGL3 than WER; setting *b* = 1 suggests the opposite. By allowing the expression of the WER-complex to be probabilistic we introduce the parameters *c_3_* (WER binding affinity) and *c_4_* (CPC binding affinity) (and *c_5_,* basal degradation), allowing us to set the two binding affinities as equal.

*Deterministic expression:*

The probabilistic functions not only allow us more control over the effects of particular component interactions and provide scope to investigate interaction strengths, but they add stochasticity to the system. This is important for simulations of the *scm* mutant (Fig. S2B and S3B). Fig. S2 shows simulation results for a completely deterministic epi-net in which the initial states of all components, bar SCM, are zero. In Fig. S2A, *WER* mRNA is dependent on cortical bias (*a* = 0); in Fig. S2B, *WER* mRNA is independent of cortical bias (*a* = 1). For the WER-complex, *b* = 1 (the active movement coded into our Boolean model means that the value of *b* has a negligible effect on the fixed point of the epi-net, as WER and CPC are rarely both equal to 1 in the same cell-net). The epi-net sensitive to cortical bias (Fig. S2A) forms distinct cell-net types in the correct position. However, the epi-net independent of cortical bias (Fig. S2B) enters a periodic (oscillatory) fixed point, and no distinct cell-net types are formed.

Random initial conditions do not supply the noise needed for the SCM-independent epi-net to form distinct cell-net types (other than in a very few, uncharacteristic cases—for example when the random initial condition happens to be the 16 distinct cell types). Generically, the SCM-independent epi-net enters a variety of periodic orbits that do not correspond to distinct cell types. Random initial conditions (retaining alternating SCM expression) have no effect on the fixed point of the SCM-influenced epi-net.

Stochasticity can be added to the deterministic epi-net using asynchronous updating [S1]: one component of the epi-net is chosen at random at each time step and updated in accordance to its Boolean function. Fig. S3 shows that asynchronous updating adds sufficient stochasticity to the system to allow the SCM independent epi-net to form distinct cell types (Fig. S3B).

**Active movement of GL3/EGL3 protein is dispensable for epidermal patterning in the Boolean formalism**

One assumption encoded in our mathematical model is that of active movement of the GL3/EGL3 and CPC proteins from the cells in which they are translated directly into neighbouring cells. To investigate the necessity of this assumption, we use a modification to Eqs. (2) (main manuscript). We study only the mutual support model, since this model better represents the biological data.

To investigate the movement of GL3/EGL3 and CPC in our model we introduce movement parameters, *M_GL3_* and *M_CPC_* (recalling that in our mathematical formalism we use a single GL3 variable to represent both GL3 and EGL3). The introduction of movement parameters necessitates that the equations representing the dynamics of GL3 and CPC expression are no longer deterministic (as their expression will remain binary). We therefore introduce new variables representing the probability of GL3 and CPC expression, with the same meaning as the probabilistic variables for WER and WER-complex in Eqs. (2).

Consider the GL3 equation from Eqs. (2):

.

Since $\text{GL}3_{j\pm1}^{t}$ is either 0 or 1, an equivalent way of writing this is

.

We shall use this form to study different mechanisms of protein movement. Incorporating the movement parameter, and introducing the new probabilistic expression variables, yields the following equations for the probability of GL3 and CPC expression:

where 0 ≤ *M_GL3_* ≤ 1 and 0 ≤ *M_CPC_* ≤ 1. Setting *M_x_* = 1 recovers the active movement mechanism used in Eqs. (2)—all translated protein is moved out of the producing cell to the neighbouring cells. When *M_x_* = 0, protein cannot leave the cell in which it is produced. With this modification, the dynamics of the epi-net are governed by the following equations:

(3)

*Active movement of GL3/EGL3 implies active movement of CPC:*

We first consider the case where the GL3/EGL3 protein is moved actively from the cells in which it is produced (i.e. *M_GL3_* = 1). In this case, CPC protein must also move actively. To see this, we note that GL3/EGL3 is produced (translated) in cells lacking the WER-complex and moves actively to neighbouring cells. Conversely, CPC is produced in cells in which the WER-complex is present. If CPC movement is not active (*M_CPC_* ≤1/3), then the probability of CPC protein expression in producing cells will be greater than that in non-producing cells (or equal, if *M_CPC_* = 1/3). Thus, the probability of CPC protein expression will be greatest in the same cells in which GL3/EGL3 accumulates, which contradicts the reported complementary patterns of GL3 and CPC proteins (see main manuscript). We thus conclude that, in the context of the Boolean model, if GL3/EGL3 moves actively, then CPC must also do so. We turn next, therefore, to a consideration of the Boolean model in which GL3/EGL3 does not move actively (*M_GL3_* ≤1/3). In this case, we consider first the model where CPC moves actively.

*Active GL3/EGL3 movement is dispensable if CPC moves actively:*

Fig. S4 shows simulated time course data for Eqs. (3) with *M_GL3_* = 0.33, *M_CPC_* = 1 (Fig. S4A) and *M_GL3_* = 0.16, *M_CPC_* = 1 (Fig. S4B) (initial conditions and *c_k_* as in the main manuscript). When *M_GL3_* = 0.33, the probability of GL3 expression tends towards uniformity in the epi-net (analogous to strong GL3/EGL3 diffusion), whereas *M_GL3_* = 0.16 results in peaks of unbound GL3 protein in the N positions (analogous to weaker diffusion). Under these conditions, the epi-net still patterns (although patterning becomes less distinct as *M_GL3_* decreases—corresponding to weaker GL3/EGL3 diffusion). Patterning results from a combination of the cortical bias and the active movement of CPC. Since the Boolean formalism does not incorporate absolute expression levels, it is not possible to determine the overall patterns on GL3/EGL3 protein accumulation (a combination of free GL3/EGL3 and GL3/EGL3 bound in complexes with WER and CPC).

Fig. S5 shows the results of a simulation of the *scm* mutant, which lacks cortical bias. In this case, the active movement of CPC alone is sufficient to pattern the epi-nets. Fig. S6 shows a simulated *cpc* mutant; again the resulting expression patterns are comparable to experimental data. These simulation results suggest that it is mechanistically sufficient for GL3/EGL3 protein to diffuse passively between cells when CPC is actively moved from the cells in which it is produced (translated), although the patterning may not be as well-defined as when both mobile proteins are actively moved.

*Passive movement of CPC is insufficient for patterning in the absence of cortical bias:*

We next explore the necessity of active movement of CPC protein in the Boolean model. As discussed above, passive CPC movement is inconsistent with active GL3/EGL3 movement. However, the possibility remains that appropriate patterning of the epidermis could occur when *both* CPC and GL3/EGL3 move passively. In this case, it is necessary to make a slight modification to the model encoded in Eqs. (3). If CPC protein moves passively, then its probability of expression will tend to be greatest in the cells in which it is produced (translated). However, this results in a logical inconsistency if (free) CPC protein represses *WER* transcription, since WER transcription would be repressed most strongly in CPC-producing cells (which are, by definition, cells in which the WER-complex is expressed). To overcome this problem, we must assume instead that it is the CPC-complex that represses *WER* transcription. We include this additional feature in our model by modifying the equation for the probability of *WER* expression such that *c_2_* is now deducted from $P_{j}^{t+1}[\text{WER}]$ if *both* CPC and GL3/EGL3 are expressed (so that CPC-complex can form):

(4)

We simulated wild type and *scm* mutant epi-nets for all four combinations of *M_GL3_* and *M_CPC_* = {0.33, 0.16}. All wild type simulations patterned appropriately because of the imposed cortical bias in *WER* transcription. In contrast, all *scm* mutant simulations failed to pattern. Fig. S7 shows an example of a wild type and *scm* mutant simulation. While these results suggest that active movement of the CPC protein is needed for *Arabidopsis* epidermal patterning they are inconclusive because the reduction in (free) protein concentration due to the sequestering of proteins into complexes is missing from this Boolean formalism. In the following section we develop and analyze an ordinary differential equation model of the mutual support model that incorporates passive diffusion of both GL3/EGL3 and CPC proteins, together with their sequestration into CPC- and WER-complexes.

**A reaction-diffusion model for root epidermal patterning**

To assess the patterning capability of the lateral inhibition with feedback (LIF) mechanism with purely diffusive movement of the CPC and GL3 proteins, we can write down a continuous mathematical representation of the mutual support (LIF) patterning network.

(5)

*G, C, W, A* and *I* represent the (protein) concentrations of GL3/EGL3, CPC, WER, WER-complex and CPC-complex respectively, in a one-dimensional space (representing a ring of epidermal cells). GL3/EGL3 and CPC proteins can diffuse, with diffusion coefficients *D_G_* and *D_C_*. GL3/EGL3, CPC and WER undergo protein degradation with linear rates *d_G_, d_C_ and d_W_*;. Degradation of the WER-complex and CPC-complex is assumed to be negligible. WER-complexes and CPC-complexes form with first order rates *k*_1_ and *k*_3_, and dissociate with rates *k*_2_ and *k*_4_, respectively. The monotonic decreasing function *P_W_*(*I*) represents the production rate of WER, regulated by the level of expression of the CPC-complex (in this diffusion model, it is necessary that CPC represses *WER* transcription via the CPC-complex, since free CPC protein will always be highest at its site of production). The rates of production of GL3/EGL3 and CPC are dependent on the amount of WER-complex, and are described by the functions *P_G_*(*A*) and *P_C_*(*A*). Since GL3/EGL3 and CPC are repressed and activated by the WER-complex, respectively, *P_G_*(*A*) and *P_C_*(*A*) are monotonic decreasing and increasing functions of *A*. In order to assess the ability of this model to generate pattern de novo, we consider the case in which there is no imposed spatial bias.

To reduce the above model to a two-component reaction diffusion system, we assume that the dynamics of WER, WER-complex and CPC-complex are fast compared to those of GL3 and CPC. This approximation would be appropriate if diffusion of GL3 and CPC were significantly slower to equilibrate than the levels of WER protein and the protein complexes. Setting the equations for WER, WER-complex and CPC-complex to equilibrium results in the following reduced system, where and :

We note that the final three algebraic equations specify *A* as a function of *C* and *G*, and so the first two equations constitute a two-component reaction-diffusion system:

(6)

The homogeneous steady states of this system are given by the solutions of *f*(*G*,*C*) = 0 and *g*(*G*,*C*) = 0. The stability of the system to spatially inhomogeneous perturbations is determined by the properties of the Jacobian matrix evaluated at the steady state:

The elements of *J* can be found by using the chain rule of differentiation:

# Given the natures of the production functions, these imply immediately that *b* > 0 and *d* < 0 for any steady state of the system. The signs of *a* and *c* depend on the sign of , which can be either positive or negative depending on the magnitude of *I* at steady state. If , then *a* < 0 and *c* > 0, which is inconsistent with spontaneous patterning [S2]. If, however, , then *c* < 0 and *a* can take either sign. Spontaneous patterning requires that *a* > 0, which holds if

.

A two-component reaction diffusion system with this form of Jacobian matrix is referred to as a cross activator-inhibitor system, and the steady state is unstable to spatially inhomogeneous perturbations if the following conditions involving the elements of the Jacobian matrix and the diffusion coefficients hold [S2]:

(7)

These conditions are all consistent with the form of Eqs. (6) above, suggesting that this model can spontaneously generate pattern with purely diffusive intercellular movement of GL3 and CPC. Furthermore, we note that for this class of system, the spatially patterned solutions that grow from the homogeneous steady state are out of phase: peaks of GL3 alternate with peaks of CPC, as observed in the root epidermis [S2].

To illustrate the capacity of this model to generate appropriate patterns of network activity, we use the following forms for the production rates of GL3, CPC and WER:

Table S4 lists a specific set of parameters that satisfy the conditions for diffusion-driven instability (Eqs. (7)) and generate patterns with a spatial periodicity of approximately two cell lengths. Numerical simulation of the reduced model (Eqs. (6)) on a spatial domain of length 16 cells with periodic boundary conditions confirms that these parameters lead to the spontaneous generation of stable spatial patterns with eight peaks of network activity. This behaviour is also exhibited by the full model described by Eqs. (5) (Fig. S8).

The model described by Eqs. (5) assumes that patterning takes place in continuous space. To show that the same LIF mechanism can also generate appropriate spatial patterns on a discrete space of 16 cells, we have simulated the model on such a space, with linear (diffusive) flux coupling between neighbouring cells. Fig. S9 shows the resulting patterns, which match the expression patterns observed experimentally in the root epidermis.

These results show that the diffusive movement of GL3 and CPC between epidermal cells, together with the feedback regulation summarised in Fig. 2B, is sufficient to generate appropriate stable spatial patterns of activity of the epidermal patterning network. The patterns generated by the diffusive LIF model depend on the amplification of small spatially-varying perturbations from the homogeneous steady state. This amplification, operating via a ‘double negative’ feedback loop, results in mutually exclusive patterns of accumulation of free GL3 and CPC protein. Generation of the observed complementary patterns of accumulation of *total* levels of GL3 and CPC protein (see main manuscript) depends on the establishment of an appropriate equilibrium between free and complexed forms of the proteins, which in turn depends on the relative magnitudes of the association and dissociation rates. Thus, the diffusion-based mechanism depends on the same regulatory logic that underlies our Boolean state model, but provides in addition an illustration of how mechanistic parameters control patterning.

# Supplementary References

S1. Harvey I, Bossomaier T (1997) Time out of joint: Attractors in asynchronous random Boolean networks. Proceedings of the Fourth European Conference on Artificial Life (ECAL97). Boston: MIT Press. pp. 67–75.

S2. Murray JD (2003) Mathematical Biology, 3rd. Edition. New York: Springer.

## Supporting Figure legends

**Figure S1:** Graphs of the data in Table S2, showing the average number of iterations needed for *WER* mRNA (solid line) and WER-complex (dashed line) to enter the alternating trichoblast/atrichoblast steady state in the mutual support model, together with the percentage of epi-nets entering the alternating steady state for varying *c_i_* (averaged over 500 simulations). The blue lines show the values of *c_i_* used in the simulations documented in Figs. 4, 5, 6, 7 and 9.

**Figure S2:** Time course graphs showing simulated data for deterministic epi-nets; (A) *WER* mRNA dependent on cortical bias (*a* = 0), (B) *WER* mRNA independent of cortical bias (*a* = 1). The initial state of all components, bar SCM, is zero. The epi-net sensitive to cortical bias (A) forms distinct cell-net types corresponding to trichoblast and atrichoblast cells in the appropriate position; however, the epi-net independent of cortical bias (B) enters a periodic fixed point and no distinct cell-net types are formed.

**Figure S3:** Time course graphs showing simulated data for deterministic epi-nets updated asynchronously [S1]; (A) *WER* mRNA dependent on cortical bias (*a* = 0), (B) *WER* mRNA independent of cortical bias (*a* = 1). Both epi-nets form distinct cell types corresponding to trichoblast and atrichoblast cells.

**Figure S4:** Simulated time course data for Eqs. (3), showing the effect on patterning of GL3/EGL3 passive movement for wild type simulations. (A) *M_GL3_* = 0.33, *M_CPC_* = 1 (B) *M_GL3_* = 0.16, *M_CPC_* = 1.

**Figure S5:** Simulated time course data for Eqs. (3), showing the effect on patterning of GL3/EGL3 passive movement for *scm* mutant simulations. (A) *M_GL3_* = 0.33, *M_CPC_* = 1 (B) *M_GL3_* = 0.16, *M_CPC_* = 1.

**Figure S6:** Simulated time course data for Eqs. (3), showing the effect on patterning of GL3/EGL3 passive movement for *cpc* mutant simulations. (A) *M_GL3_* = 0.33, *M_CPC_* = 1 (B) *M_GL3_* = 0.16, *M_CPC_* = 1.

**Figure S7:** Simulated time course data for Eqs. (4), showing the effect on patterning of GL3/EGL3 and CPC passive movement. (A) Wild type simulation with *M_GL3_* = 0.16, *M_CPC_* = 0.16 (B) *scm* mutant simulation with *M_GL3_* = 0.16, *M_CPC_* = 0.16.

**Figure S8:** Simulated time course data for the continuous space diffusion model defined by Eqs. (5). The model was simulated on a spatial grid with ten spatial points per cell length (a total of 160 spatial points) and using a standard finite difference scheme for the diffusion term with periodic boundary conditions. The resulting set of ordinary differential equations was simulated using the Matlab Runge-Kutta solver ode23. The parameters used are listed in Table S4.

**Figure S9:** Simulated time course data for the model defined by Eqs, (5) using a spatial grid with one spatial point per cell length (a total of 16 spatial points). All other simulation details were as in Figure S8.

## Supporting Table legends

**Table S1:** States and probabilities of expression for network components in the H and N positions when the epi-net is in the alternating steady state.

**Table S2:** Average number of iterations needed for *WER* and WER-complex to reach the alternating trichoblast/atrichoblast steady state in the mutual support model (shown in Table S1 and Fig. 3B), averaged over 500 simulations, rounded to the nearest whole number. The values of *c_i_* used for the simulations in Figs. 4, 5, 6, 7 and 9 are shown in bold type. UU0 in the table indicates that the alternating steady state was not reached for any of the 500 simulations. Superscripts denote epi-nets corresponding to specific experimental conditions: ^*^ *scm* mutant, ^**^ *wer* mutant, ^+^ WER over-expression, ^++^ *cpc, ttg, gl3 egl3* mutants.

**Table S3:** Percentage of simulations (s.s. (%)) which attain the alternating steady state with different initial probabilities of expression for *WER* and WER-complex. Percentages were determined from 500 independent simulations for each set of initial probabilities, rounded to one decimal place. The initial probabilities used for the simulations in Figs. 4, 5, 6, 7 and 9 are shown in bold type.

**Table S4:** Example parameter values for which the model defined by Eqs. (5) generates stable spatial patterns via diffusion-driven instability. Units for each parameter are given in terms of protein concentration (conc.), time and the length of each epidermal cell (cell length).

# Supporting Figures

# Figure S1

#
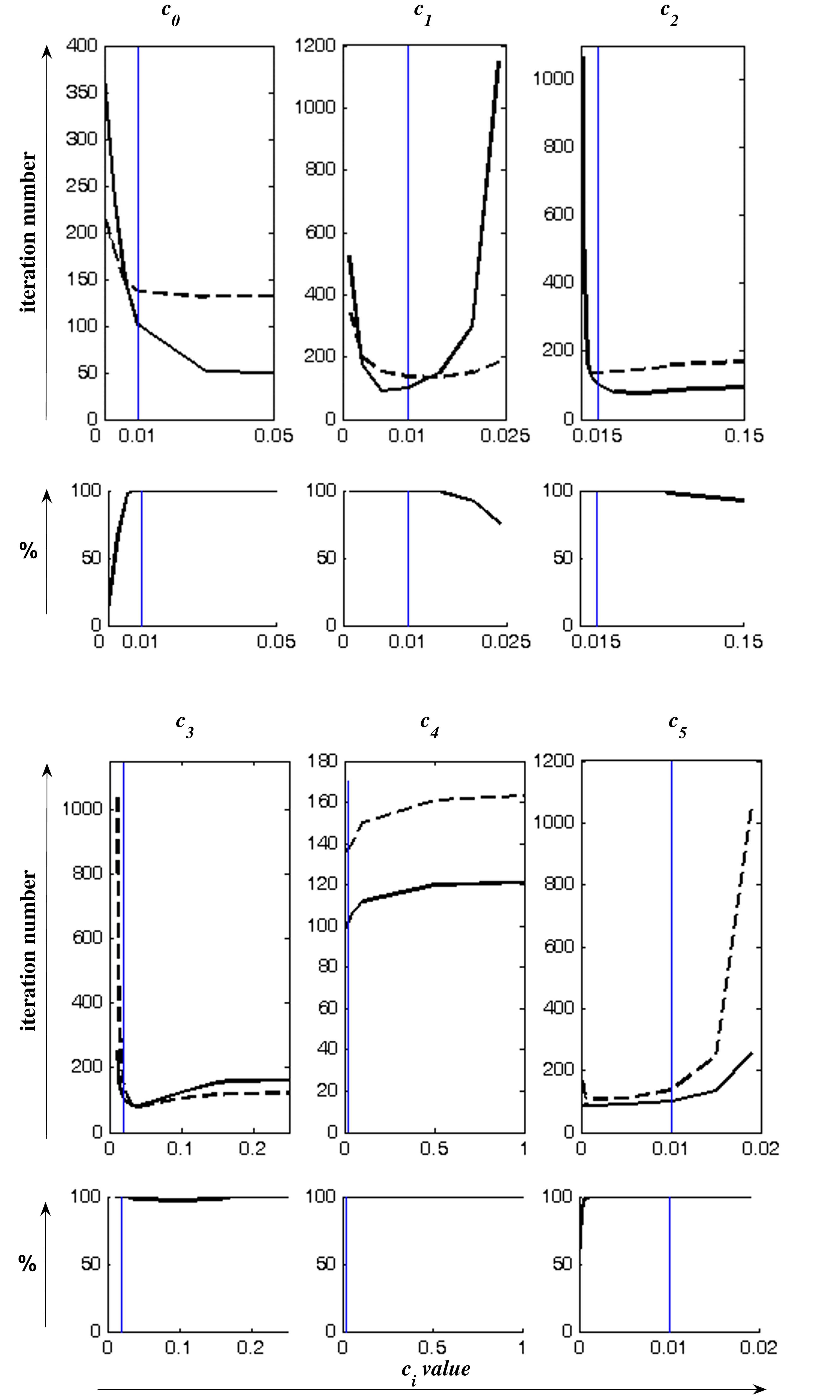


# Figure S2

#
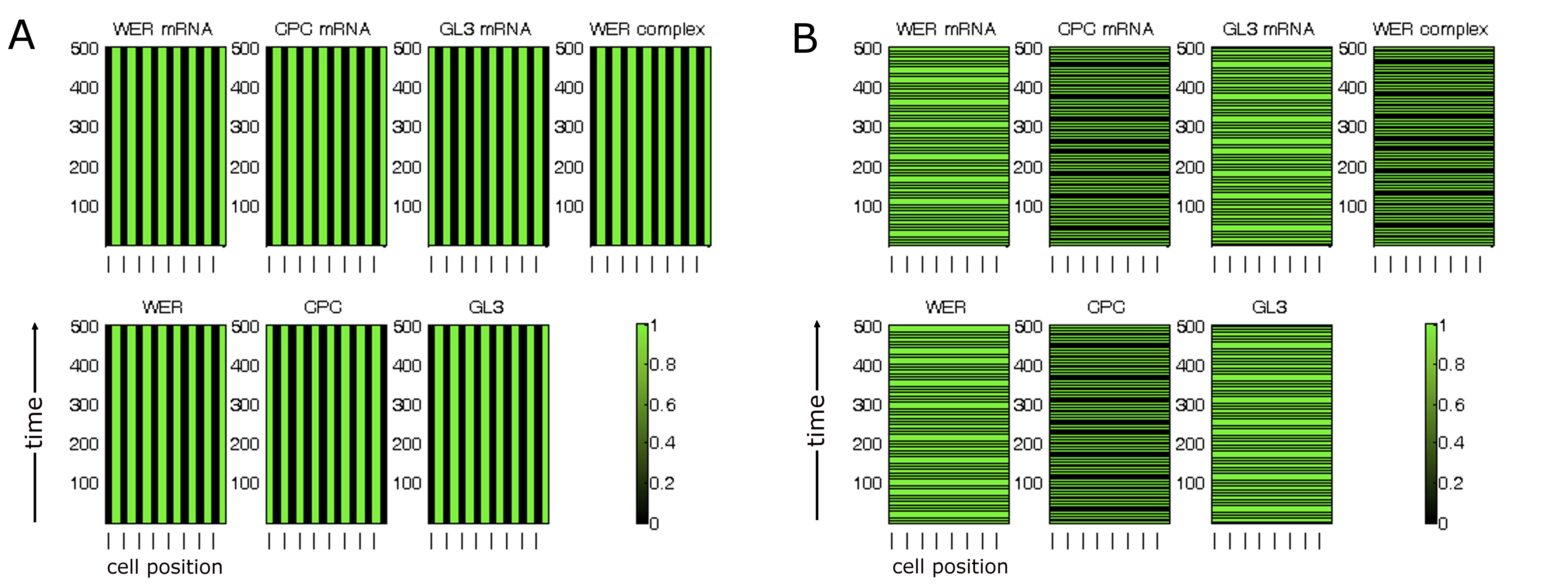


# Figure S3

#
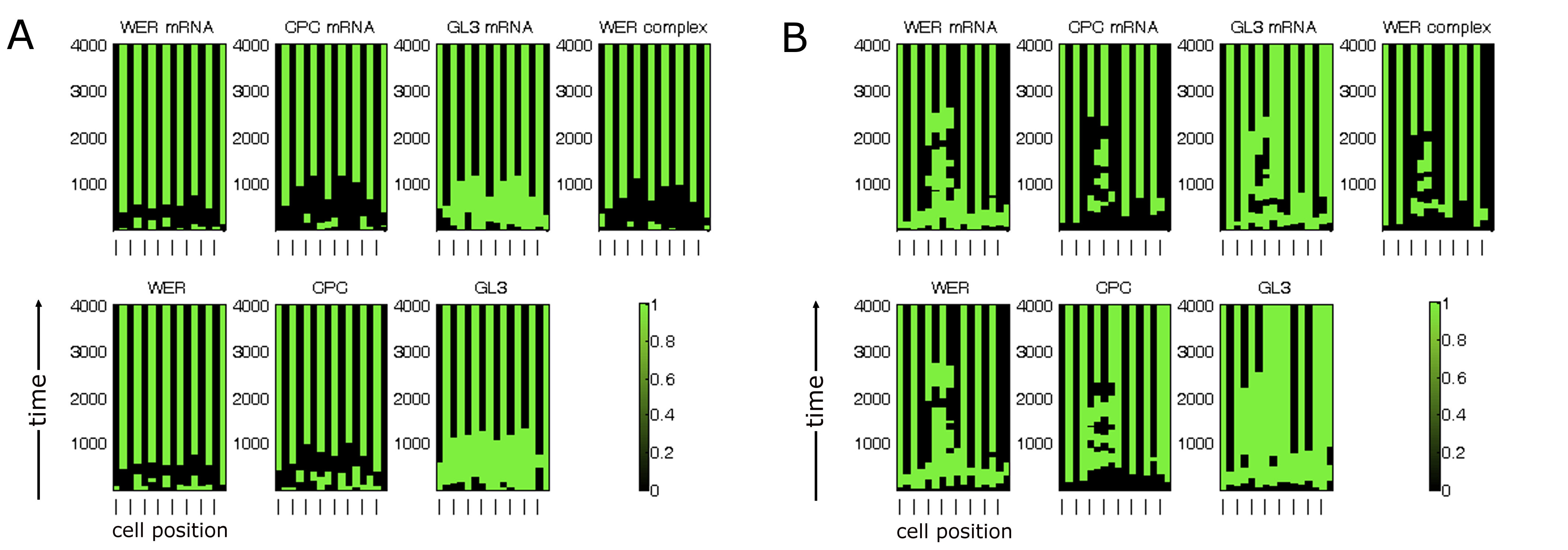


# Figure S4

#
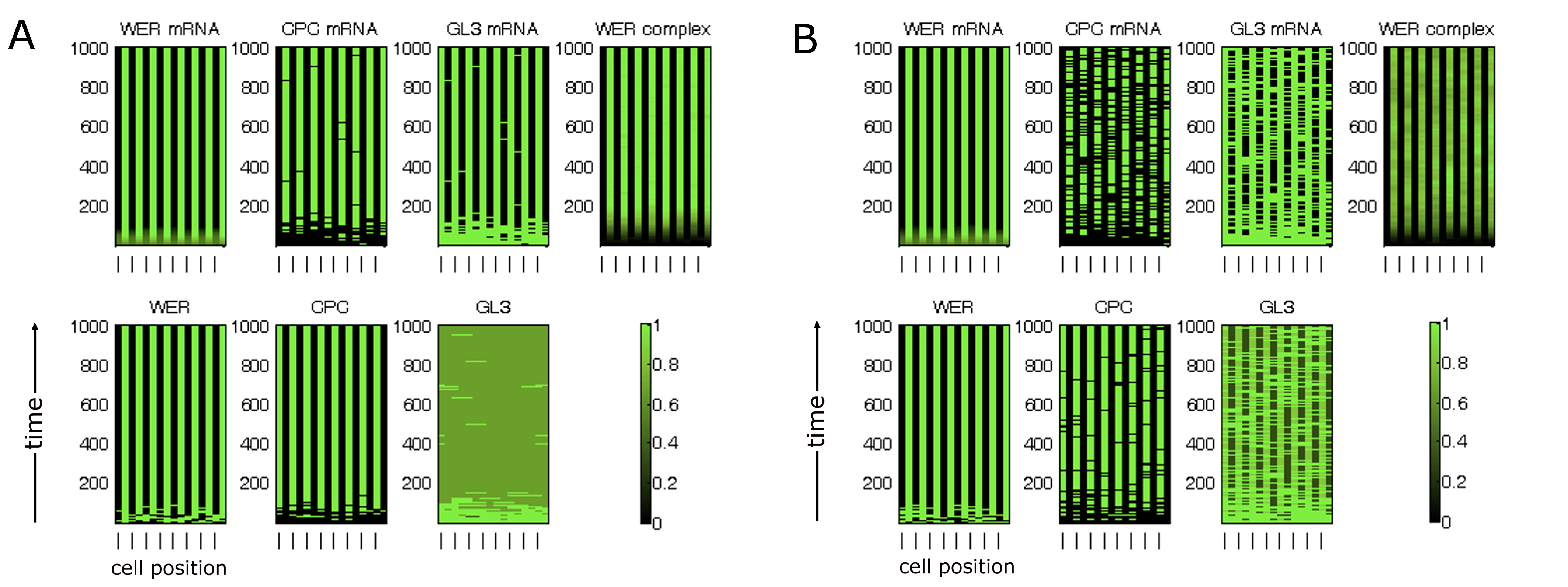


# Figure S5

#
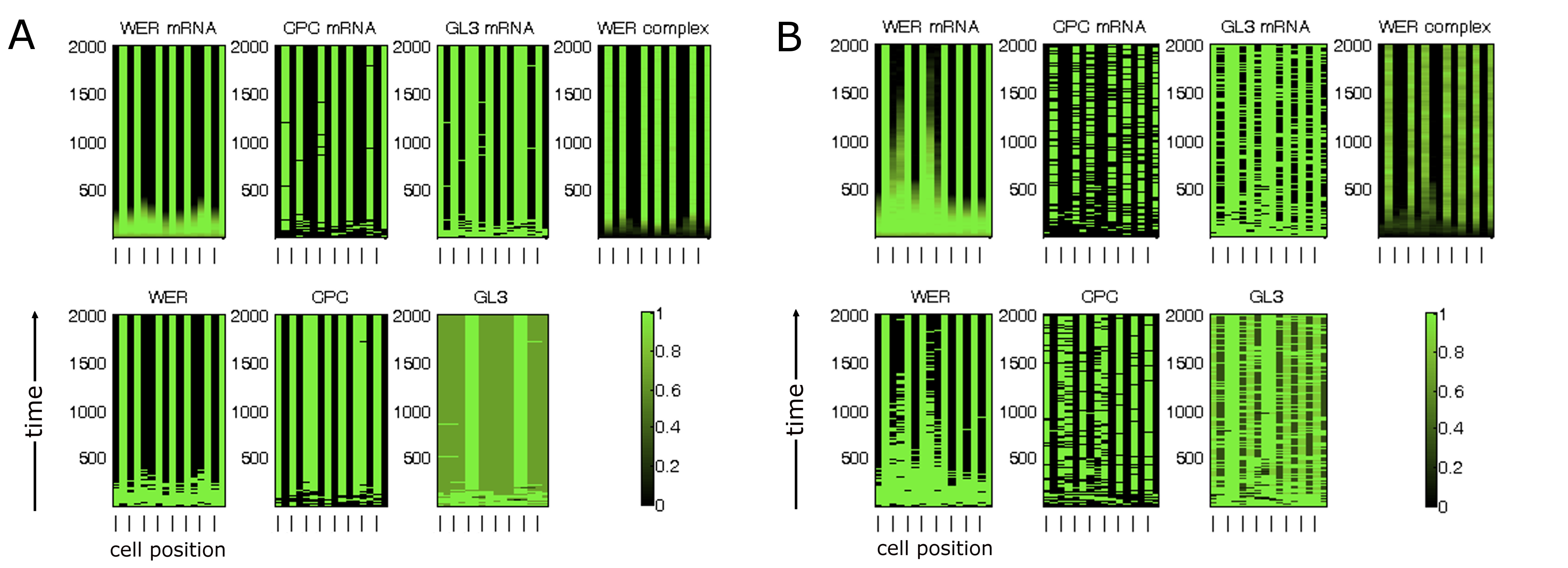


# Figure S6

#
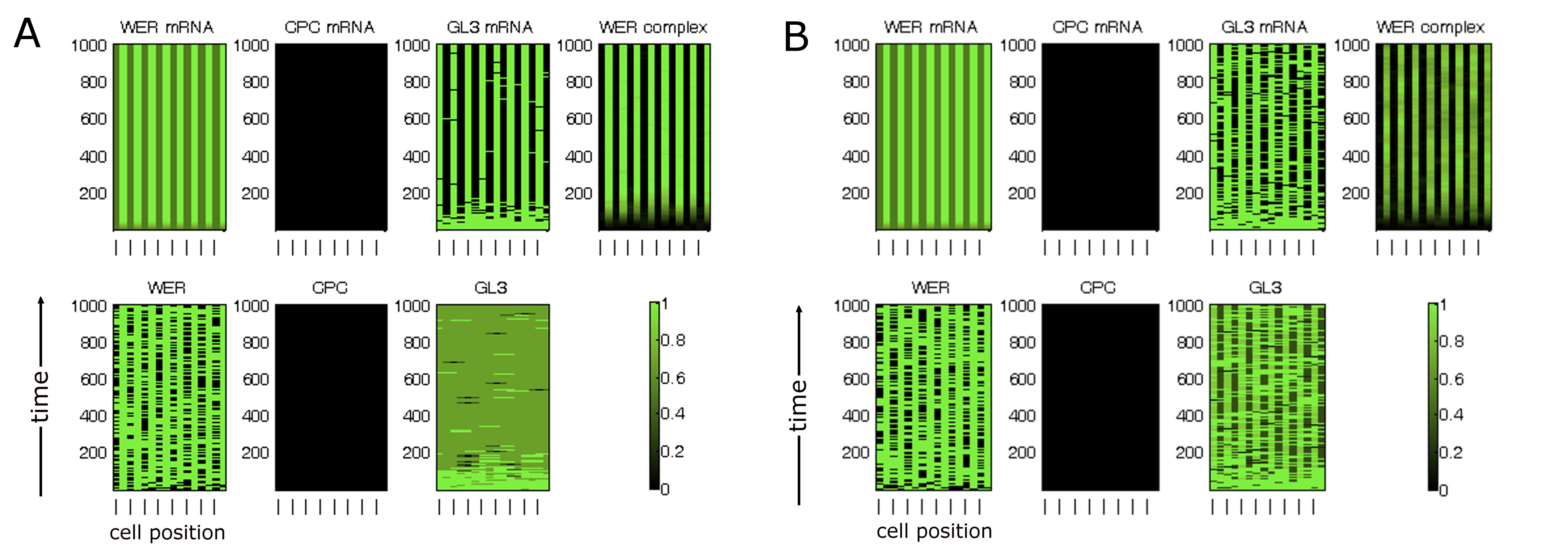


# Figure S7

#
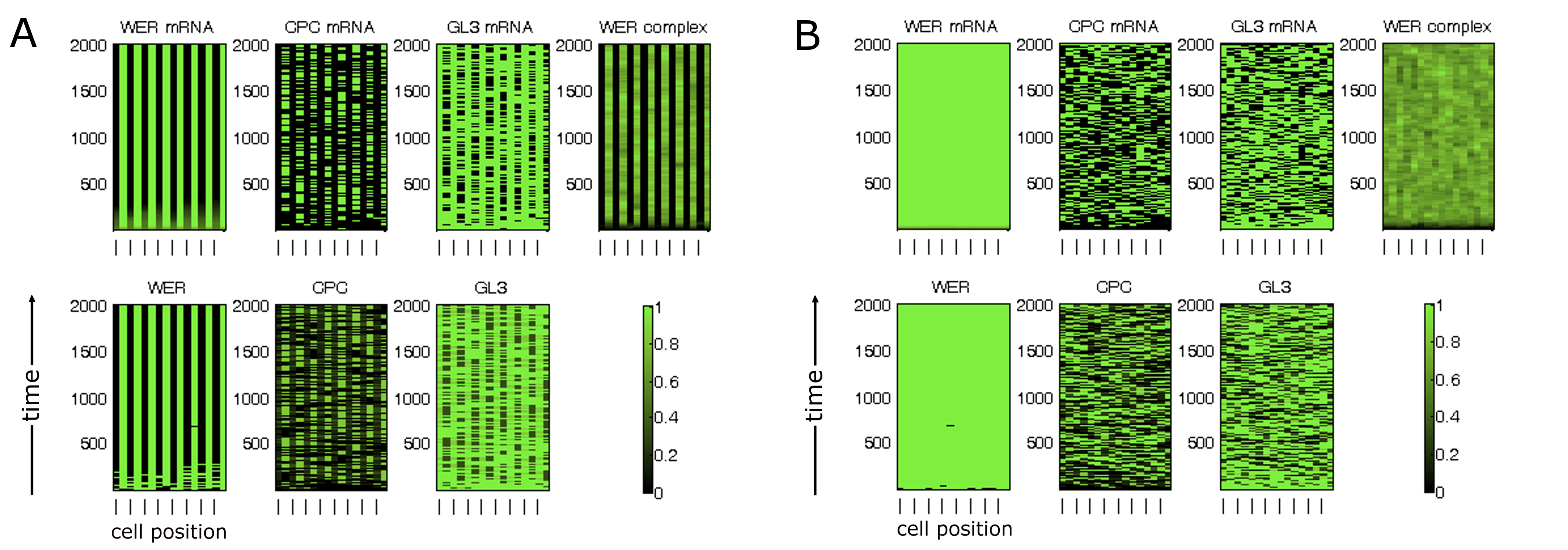


# Figure S8

#
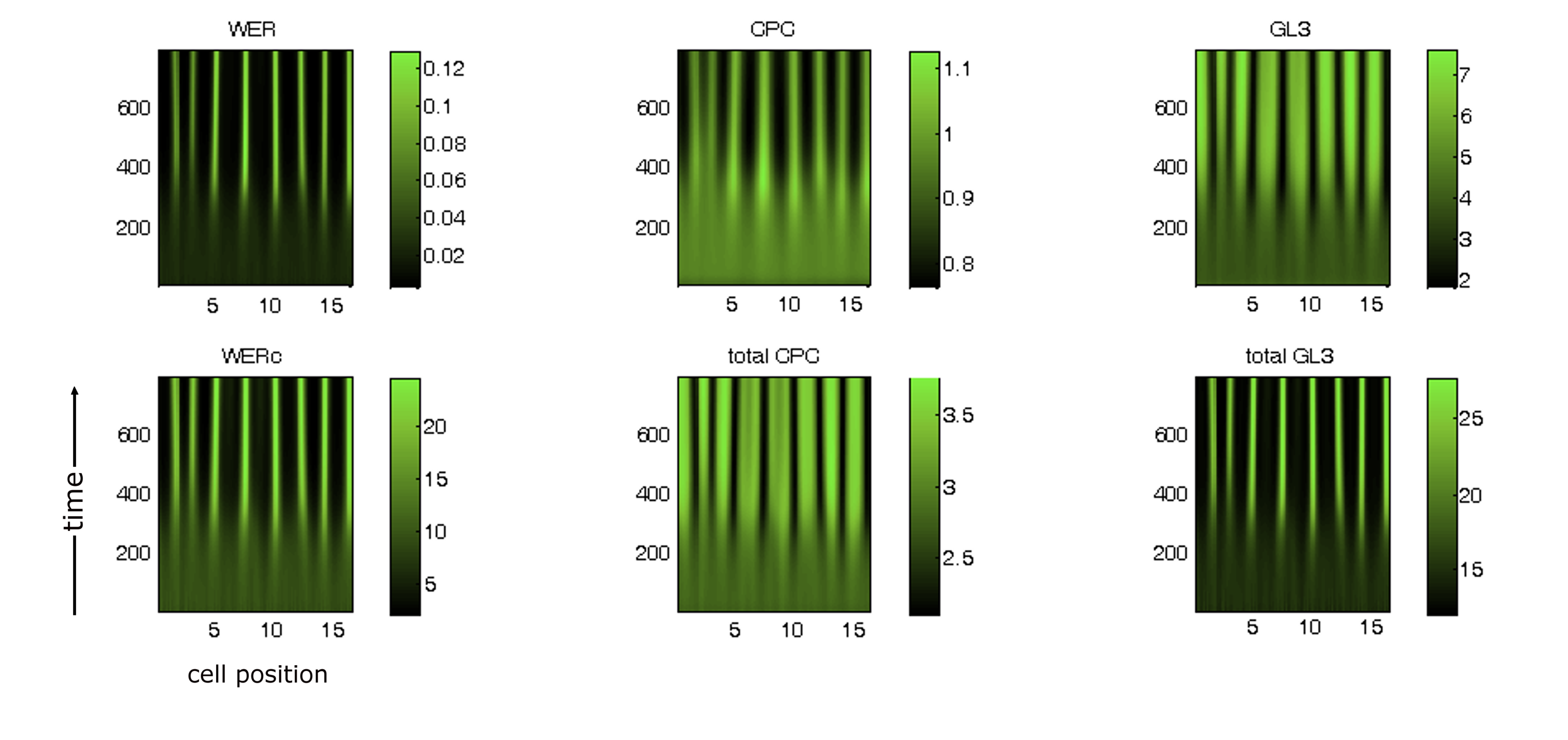


# Figure S9

#
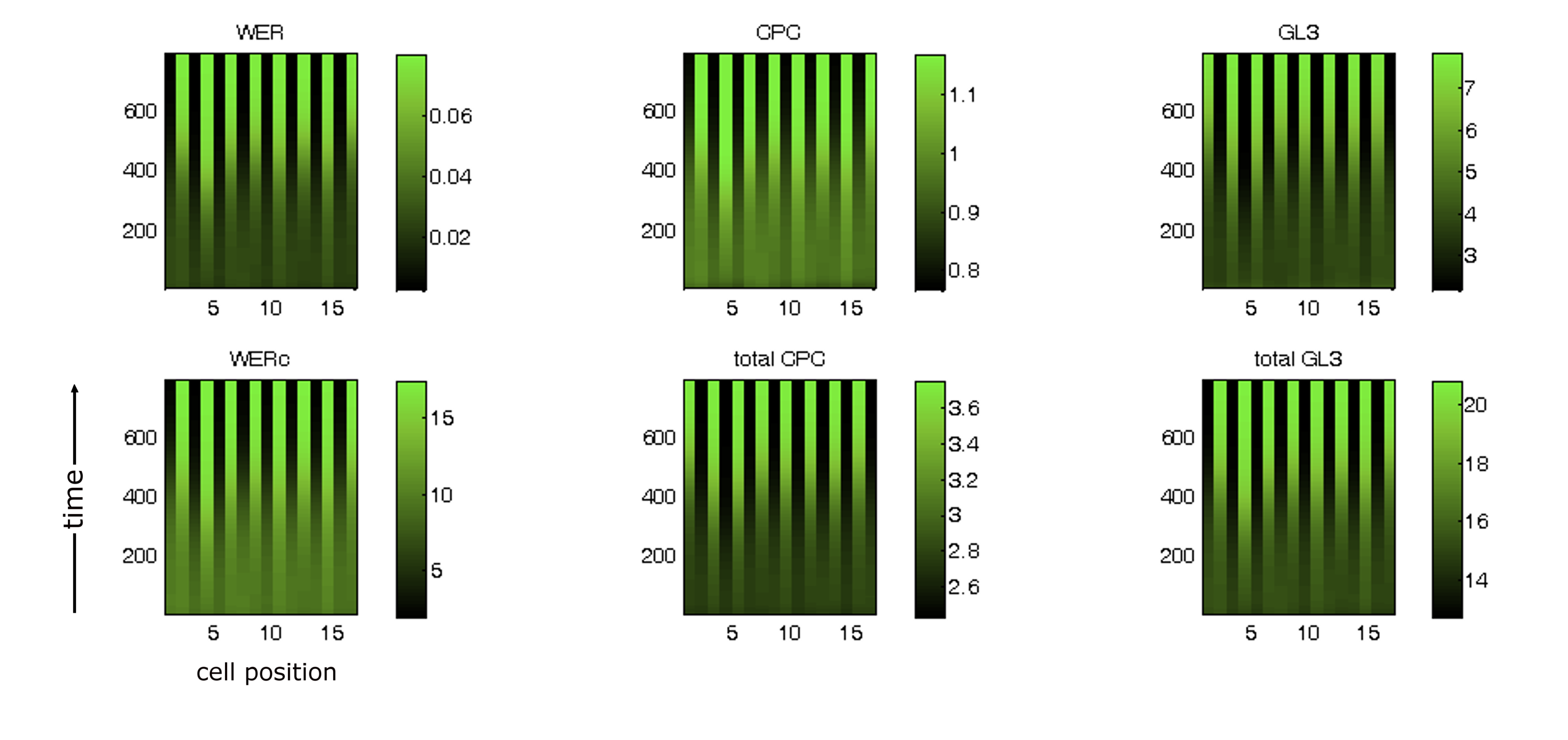


# Supporting Tables

|  |  | SCM | *WER* | WERp | *GL3* | GL3p | WERc | *CPC* | CPCp |
| --- | --- | --- | --- | --- | --- | --- | --- | --- | --- |
| H | state | 1 | 0 | 0 | 1 | 0 | 0 | 0 | 1 |
|  | probability | - | 0.0 | - | - | - | 0.0 | - | - |
| N | state | 0 | 1 | 1 | 0 | 1 | 1 | 1 | 0 |
|  | probability | - | 1.0 | - | - | - | 1.0 | - | - |

Table S1: States and probabilities of expression for network components in the H and N positions when the epi-net is in the alternating steady state.

| *c*_0_ |  |  | 0^*^ | .003 | .006 | .007 | **.01** | .03 | .05 | 1 |  |  |  |  |
| --- | --- | --- | --- | --- | --- | --- | --- | --- | --- | --- | --- | --- | --- | --- |
| iteration  number | *WER* |  | 368 | 234 | 153 | 136 | **101** | 51 | 50 | 49 |  |  |  |  |
|  | WERc |  | 220 | 180 | 150 | 145 | **137** | 131 | 131 | 130 |  |  |  |  |
| s.s. (%) | |  | 11 | 67 | 99 | 100 | **100** | 100 | 100 | 100 |  |  |  |  |
| *c*_1_ |  |  | 0^**^ | .001 | .003 | .006 | **.01** | .015 | .02 | .024 | .025^+^ |  |  |  |
| iteration  number | *WER* |  | **U** | 526 | 176 | 90 | **101** | 148 | 300 | 1151 | **U** |  |  |  |
|  | WERc |  | **U** | 360 | 200 | 154 | **137** | 134 | 151 | 183 | **U** |  |  |  |
| s.s. (%) | |  | **0** | 100 | 100 | 100 | **100** | 100 | 93 | 76 | **0** |  |  |  |
| *c*_2_ |  | 0^++^ | .0005 | .001 | .005 | .01 | **.015** | .03 | .05 | .08 | .09 | .5 | 1 |  |
| iteration  number | *WER* | **U** | 1068 | 568 | 168 | 119 | **101** | 81 | 76 | 80 | 86 | 131 | 169 |  |
|  | WERc | **U** | 136 | 136 | 136 | 137 | **137** | 140 | 146 | 154 | 161 | 208 | 246 |  |
| s.s. (%) | | **0** | 100 | 100 | 100 | 100 | **100** | 100 | 100 | 100 | 99 | 67 | 65 |  |
| *c*_3_ |  | .01^++^ | .011 | .012 | .013 | .016 | **.02** | .03 | .04 | .1 | .15 | .17 | .5 | 1 |
| iteration  number | *WER* | **U** | 254 | 191 | 162 | 123 | **101** | 83 | 81 | 135 | 155 | 159 | 167 | 168 |
|  | WERc | **U** | 1057 | 549 | 379 | 207 | **137** | 91 | 80 | 106 | 118 | 119 | 127 | 129 |
| s.s. (%) | | **0** | 100 | 100 | 100 | 100 | **100** | 100 | 99 | 98 | 99 | 100 | 100 | 100 |
| *c*_4_ |  |  |  | 0 | .005 | .01 | **.02** | .03 | .1 | .5 | 1 |  |  |  |
| iteration  number | *WER* |  |  | 98 | 99 | 100 | **101** | 104 | 112 | 120 | 121 |  |  |  |
|  | WERc |  |  | 133 | 135 | 136 | **137** | 139 | 150 | 161 | 163 |  |  |  |
| s.s. (%) | |  |  | 100 | 100 | 100 | **100** | 100 | 100 | 100 | 100 |  |  |  |
| *c*_5_ |  | 0 | .0001 | .0005 | .001 | .005 | **.01** | .015 | .019 | .02^++^ |  |  |  |  |
| iteration  number | *WER* | 89 | 87 | 88 | 87 | 90 | **101** | 135 | 255 | **U** |  |  |  |  |
|  | WERc | 101 | 179 | 133 | 108 | 109 | **137** | 244 | 1057 | **U** |  |  |  |  |
| s.s. (%) | | 50 | 73 | 99 | 100 | 100 | **100** | 100 | 100 | **0** |  |  |  |  |

Table S2: Average number of iterations needed for *WER* and WER-complex to reach the alternating trichoblast/atrichoblast steady state in the mutual support model (shown in Table S1 and Fig. 3B), averaged over 500 simulations, rounded to the nearest whole number. The values of *c_i_* used for the simulations in Figs. 4, 5, 6, 7 and 9 are shown in bold type. UU0 in the table indicates that the alternating steady state was not reached for any of the 500 simulations. Superscripts denote epi-nets corresponding to specific experimental conditions: ^*^ *scm* mutant, ^**^ *wer* mutant, ^+^ WER over-expression, ^++^ *cpc, ttg, gl3 egl3* mutants.

| *WER* | 0 | 0 | 0 | **0.5** | 0.5 | 0.5 | 1 | 1 | 1 |
| --- | --- | --- | --- | --- | --- | --- | --- | --- | --- |
| WERc | 0 | 0.5 | 1 | **0** | 0.5 | 1 | 0 | 0.5 | 1 |
| s.s. (%) | 100 | 100 | 100 | **100** | 100 | 100 | 80.2 | 99.4 | 100 |

Table S3: Percentage of simulations (s.s. (%)) which attain the alternating steady state with different initial probabilities of expression for *WER* and WER-complex. Percentages were determined from 500 independent simulations for each set of initial probabilities, rounded to one decimal place. The initial probabilities used for the simulations in Figs. 4, 5, 6, 7 and 9 are shown in bold type.

| *p_G_* | 1 conc./time | *k*_1_ | 10 /conc. time |
| --- | --- | --- | --- |
| *p_C_* | 0.01 /time | *k*_2_ | 0.1 /time |
| *p_W_* | 1 conc./time | *k*_3_ | 5 /conc. time |
| *μ* | 0.1 /conc. | *k*_4_ | 10 /time |
| *ν* | 2 /conc. | *d_C_* | 0.1 /time |
| *D_G_* | 0.0035 (cell length)2/time | *d_G_* | 0.1 /time |
| *D_C_* | 0.07 (cell length)2/time | *d_W_* | 1 /time |

**Table S4:** Example parameter values for which the model defined by Eqs. (5) generates stable spatial patterns via diffusion-driven instability. Units for each parameter are given in terms of protein concentration (conc.), time and the length of each epidermal cell (cell length).
